# Genetic control of the root system in rice under normal and drought stress conditions by genome-wide association study

**DOI:** 10.1371/journal.pgen.1006889

**Published:** 2017-07-07

**Authors:** Xiaokai Li, Zilong Guo, Yan Lv, Xiang Cen, Xipeng Ding, Hua Wu, Xianghua Li, Jianping Huang, Lizhong Xiong

**Affiliations:** National Key Laboratory of Crop Genetic Improvement and National Center of Plant Gene Research (Wuhan), Huazhong Agricultural University, Wuhan, China; Shanghai Institutes for Biological Sciences, CHINA

## Abstract

A variety of adverse conditions including drought stress severely affect rice production. Root system plays a critical role in drought avoidance, which is one of the major mechanisms of drought resistance. In this study, we adopted genome-wide association study (GWAS) to dissect the genetic basis controlling various root traits by using a natural population consisting of 529 representative rice accessions. A total of 413 suggestive associations, containing 143 significant associations, were identified for 21 root traits, such as maximum root length, root volume, and root dry weight under normal and drought stress conditions at the maturation stage. More than 80 percent of the suggestive loci were located in the region of reported QTLs for root traits, while about 20 percent of suggestive loci were novel loci detected in this study. Besides, 11 reported root-related genes, including *DRO1*, *WOX11*, and *OsPID*, were found to co-locate with the association loci. We further proved that the association results can facilitate the efficient identification of causal genes for root traits by the two case studies of *Nal1* and *OsJAZ1*. These loci and their candidate causal genes provide an important basis for the genetic improvement of root traits and drought resistance.

## Author summary

Drought stress is a key environmental factor that severely reduces crop yield all over the world. The root system plays a critical role in the drought avoidance of crops, but the genetic basis of the root system in soil conditions has seldom been investigated in rice. We analyzed the genetic control of 21 root traits via genome-wide association study (GWAS) of a natural population, and we identified 110 significant association loci containing many reported and unknown candidate genes related to root development. We presented the case studies of two genes, *Nal1* and *OsJAZ1*, to demonstrate the high efficiency of the identification of genes in the control of root traits based on the association and candidate gene analyses. Our results would provide a foundation not only for elucidating the genetic and molecular basis of root development but also for improving the drought avoidance of rice.

## Introduction

Rice (*Oryza sativa* L.) is the staple food crop that feeds a large segment of the world’s population [1]. Owing to the climate change and shortage of freshwater, drought has been the most critical environmental stress influencing agriculture worldwide, particularly regarding the productivity of field crops [2], especially for rice. To improve the drought resistance of rice and increase its yield under drought stress conditions is of great significance.

Plant roots play an important role in the absorption and translocation of water and nutrients. The vibrant root system would allow crops to gain more water, and improvement of the root system architecture will contribute to drought avoidance in crops [3]. Some root traits are associated with plant productivity under drought stress conditions, such as fine root diameters, specific root length, and considerable root density [4]. In recent decades many researchers have tried to uncover the genetic basis of root traits in rice, aiming to improve the drought resistance of rice and to increase its yield under drought stress conditions.

Traditionally, linkage mapping has been commonly employed to detect quantitative trait loci (QTL) for complex traits, including root architecture traits. In 1990s, linkage mapping of root traits in rice was conducted for the first time by using a population of 203 recombinant inbred lines (RILs) which were treated with drought stress at different stages, and five parameters of root morphology were investigated [5]. Since then, many QTLs related to root traits, such as root penetration ability, root growth rate under drought stress conditions, deep root morphology, and root thickness, were identified by linkage mapping in different populations [6–8]. A critical study on linkage mapping were conducted for drought resistance-related root traits, fitness, and productivity related traits in a population of 180 RILs, and 36 and 38 QTLs for different root traits were identified under normal and drought stress conditions, respectively [9]. In a subsequent study, a QTL controlling root volume in rice, *qFSR4*, was fine mapped to a region of 38-kb on chromosome 4, and *Narrow leaf 1* (*Nal1*) has been assumed to be the candidate gene for this QTL [10]. In another RIL population, 84 additive-effect QTLs and 86 pairs of epistatic QTLs were detected for six root traits at five different stages [11]. Meanwhile, several root related QTLs in rice have been verified in the past few years [12]. *Sta1*, a QTL controlling the stele transversal area in root of rice, was fine mapped to a 359-kb interval on chromosome 9 [13]. *(DEEPER ROOTING 1) DRO1* [14], *DRO2* [15], and *DRO3* [16], three major QTLs in the control of deep rooting of rice, were identified under normal conditions using a basket method; *qSOR1*, a major QTL controlling the soil-surface rooting of rice in paddy fields, was mapped to an 812-kb interval on the long arm of chromosome 7 [17]; *qRL6*.*1* [18] and *qRL7* [19], two major QTLs associated with the root length of rice, were identified in hydroponic conditions. Remarkably, the causal gene of the QTL *DRO1* has been cloned, and *DRO1* is involved in the regulation of deep rooting by affecting root growth angle [3].

The next-generation sequencing technology coupled with the growing number of genome sequences opens the opportunity to redesign genotyping strategies for more effective genetic mapping and genome analysis [20]. In recent years, genome-wide association study (GWAS) has been widely used as a powerful tool to reconnect a trait back to its underlying genetics [21].

Several association studies have been conducted for root traits in rice and other crops. The genetic architecture of aluminum tolerance in rice was analyzed through GWAS and bi-parental linkage mapping of the relative root growth of the total root system [22]. A GWAS for root related traits was carried out in a panel of 167 *japonica* rice accessions by using a hydroponic cultivation system at the seedling stage in rice [23]. Another GWAS involving the ratio of deep rooting by a modified ‘basket’ method in the field was performed in a population of 237 rice varieties, coupled with linkage mapping for the same trait in 180 recombinant inbred lines [24]. In maize, 268 marker-trait associations were detected in a GWAS for 22 seedling root architecture traits using 384 inbred lines [25]. In barley, 11 putative QTL for root related traits were found in a GWAS using a unique diversity set [26]. However, considering the complexity and plasticity of root development in crops, the genetic basis of root traits at the adult and seed maturation stages, especially for a population grown in soil under drought stress conditions, remains to be elucidated.

In this study, the genetic architecture of root traits at the seed maturation stage under normal and drought stress conditions was investigated by GWAS using a panel of 529 rice accessions collected worldwide. We adopted a protocol for drought treatment by planting and stressing rice plants grown in individual polyvinyl chloride (PVC) tubes in which each genotype was stressed to the same extent at the same developmental stage [9]. The results showed that 225 of 264 loci identified by GWAS overlapped with reported root related QTLs and 11 reported root related genes were located in the corresponding region. In addition, two candidate genes, *Nal1* and *OsJAZ1*, identified by association analysis, were confirmed to control the corresponding root traits by genetic experiments. These results revealed a complete genetic control of the root system in rice at the reproductive stage under both normal and drought stress conditions, which could be an important basis for the genetic improvement of root traits and drought resistance.

## Results

### Phenotypic variation of root traits under normal and drought stress conditions

In order to systematically dissect the genetic basis of root traits of rice grown in soil, a natural population containing 529 diverse rice accessions [27] were evaluated for various root traits under normal and drought stress conditions in PVC tubes. The population exhibited a distinctive population structure and was mainly classified into *indica* subpopulation (295 accessions) including *ind I*, *ind II*, and *indica* intermediate (*ind*), and *japonica* subpopulation (156 accessions) including *tej*, *trj*, and *japonica* intermediate (*jap*) (S1 Table).

Drought stress was applied at the booting stage, and the plants were recovered when all of the leaves became fully rolled. A total of 21 root related traits, such as maximum root length under normal (MRLN) and drought stress conditions (MRLD), volume of the deep roots (> 30 cm) under normal (RVDN) and drought stress conditions (RVDD), and dry weight of the deep roots (> 30 cm) under normal (RWDN) and drought stress conditions (RWDD), were measured at the seed maturation stage (Table 1). These traits were classified into four categories: root length, root volume, root weight, and deep root rate. For most of the traits, a large range of variation was detected, with the coefficients of variation (CV) varying from 0.236 for MRLN to 1.643 for RVDD. Most of the root traits showed a normal distribution (S1 Fig) while RVDD, RVDN, RWDN and RWDD showed skewed distributions, mainly due to the presence of accessions with very short roots (< 30 cm).

**Table 1:** Phenotypic variations of 21 root traits in the 529 rice accessions.

Trait name	Trait definition	Categories	Range	Mean	SD	CV
MRLN	Maximum root length (cm) under normal conditions	root length	14.5–83.5	48.8	11.5	0.236
MRLD	Maximum root length (cm) under drought stress	root length	8–119	49.2	17.1	0.348
DLRN	Deep root length rate under normal conditions	deep root rate	0.02–0.64	0.38	0.13	0.342
DLRD	Deep root length rate under drought stress	deep root rate	0.02–0.75	0.41	0.15	0.366
MRLR	Maximum root length ratio (drought stress versus normal)	root length	0.23–2.6	1	0.3	0.300
RVSN	Volume (mL) of shallow (≤ 30 cm) roots under normal conditions	root volume	0.4–133.5	27.8	20	0.719
RVSD	Volume (mL) of shallow (≤ 30 cm) roots under drought stress	root volume	0.2–90	20.4	17.1	0.838
RVDN	Volume (mL) of deep (> 30 cm) roots under normal conditions	root volume	0–35	3.2	4.9	1.531
RVDD	Volume (mL) of deep (> 30 cm) roots under drought stress	root volume	0–32	2.8	4.6	1.643
RVTN	Volume (mL) of total roots under normal conditions	root volume	0.4–165.5	32.2	23.3	0.724
RVTD	Volume (mL) of total roots under drought stress	root volume	0.2–125	24.3	20.9	0.860
DVRN	Deep root volume rate under normal conditions	deep root rate	0.01–0.33	0.08	0.05	0.625
DVRD	Deep root volume rate under drought stress	deep root rate	0–0.5	0.07	0.07	1.000
RWSN	Dry weight (g) of shallow (≤ 30 cm) roots under normal conditions	root weight	0.08–32.35	4.74	3.74	0.789
RWSD	Dry weight (g) of shallow (≤ 30 cm) roots under drought stress	root weight	0.03–21.3	3.48	2.87	0.825
RWDN	Dry weight (g) of deep (> 30 cm) roots under normal conditions	root weight	0–11.75	0.62	0.94	1.516
RWDD	Dry weight (g) of deep (> 30 cm) roots under drought stress	root weight	0–7.35	0.52	0.73	1.404
RWTN	Dry weight (g) of total roots under normal conditions	root weight	0.08–32.65	5.73	4.39	0.766
RWTD	Dry weight (g) of total roots under drought stress	root weight	0.05–28.65	4.31	3.48	0.807
DWRN	Deep root weight rate under normal conditions	deep root rate	0–0.72	0.08	0.07	0.875
DWRD	Deep root weight rate under drought stress	deep root rate	0–0.87	0.09	0.1	1.111

Correlation analysis among the 21 traits suggests that many traits were correlated (S2 Fig), and the correlation coefficients between several trait pairs were very high, such as trait pairs of MRLN and DVRN, RVSN and RWSN, and RVSD and RWSD. Most of the traits under drought stress treatment were correlated with the same trait under normal conditions (with a correlation coefficient *r* > 0.5), such as the pair of MRLD and MRLN (*r* = 0.63), RVTD and RVTN (*r* = 0.76), and the pair of RWTD and RWTN (*r* = 0.70). In order to examine the effect of drought stress treatment on root growth without the interference of correlation, we compared the three deep root rate traits, including deep root length rate (DLR), deep root volume rate (DVR), and deep root weight rate (DWR), under normal and drought stress conditions (Table 2). All the three traits were increased significantly in the whole population after drought stress treatment. The result is consistent with the conclusion that drought stress may promote the growth of deep roots [3, 4]. However, the effect of drought stress on root growth varied in different subpopulations. For example, DWR was significantly increased after drought stress treatment in the *japonica* subpopulation but not in the *indica* subpopulation. While in different *japonica* subpopulations and different *indica* subpopulations [28], the effect of drought stress treatment was complex. This result implies that the genetic basis of root traits in different subpopulations may vary significantly. In order to reduce the effect of population structure, a mixed linear model (FaST-LMM) [29] was used for the following association study.

**Table 2:** Phenotype comparison between normal and drought stress conditions.

**Traits**	**Whole**				***indica***				***japonica***			
	N	D	D/N	*P-*value	N	D	D/N	*P-*value	N	D	D/N	*P-*value
DLR	0.38	0.41	1.09	**1.27×10**^**−3**^	0.37	0.33	0.90	**0.0107**	0.36	0.32	0.89	0.0967
DVR	0.08	0.10	1.21	**1.45×10**^**−3**^	0.07	0.07	0.93	0.4123	0.08	0.07	0.83	0.1419
DWR	0.09	0.12	1.31	**2.28×10**^**−5**^	0.08	0.08	1.04	0.6059	0.07	0.10	1.37	**0.0392**
**Traits**	***ind***				***ind I***				***ind II***			
	N	D	D/N	*P-*value	N	D	D/N	*P-*value	N	D	D/N	*P-*value
DLR	0.39	0.35	0.88	0.1030	0.36	0.30	0.85	**0.0478**	0.36	0.35	0.95	0.5006
DVR	0.09	0.09	1.02	0.8801	0.07	0.06	0.86	0.3748	0.07	0.06	0.90	0.4088
DWR	0.09	0.10	1.13	0.4358	0.07	0.07	1.04	0.7917	0.08	0.08	0.96	0.7589
**Traits**	***jap***				***tej***				***Trj***			
	N	D	D/N	*P-*value	N	D	D/N	*P-*value	N	D	D/N	*P-*value
DLR	0.39	0.36	0.94	0.6816	0.32	0.25	0.77	**0.0122**	0.45	0.48	1.08	0.3544
DVR	0.09	0.05	0.55	**0.0414**	0.07	0.05	0.74	0.1386	0.11	0.12	1.10	0.5894
DWR	0.12	0.11	0.89	0.6983	0.05	0.08	1.56	0.1099	0.10	0.14	1.43	**0.0229**

N, normal conditions; D, drought stress conditions; D/N, the ratio of the value under normal conditions versus the value under drought stress conditions. *P*-values in bold represent significant difference.

### Association analysis of root traits under normal and drought stress conditions

To identify root trait related association loci, we used a reported genotypic dataset consisting of about 6.4 million SNPs generated for the 529 accessions [27] to conduct GWAS for the 21 root related traits. Using a Bonferroni correction based on the effective numbers of independent markers [30], the *P*-value thresholds were set at 1.21×10^−6^ (suggestive) and 6.03×10^−8^ (significant). In our study, a total of 413 suggestive associations with 373 lead SNPs were identified, and among them 143 associations (133 significant SNPs) exceeded the significant threshold (S2 Table).

**Fig 1:**
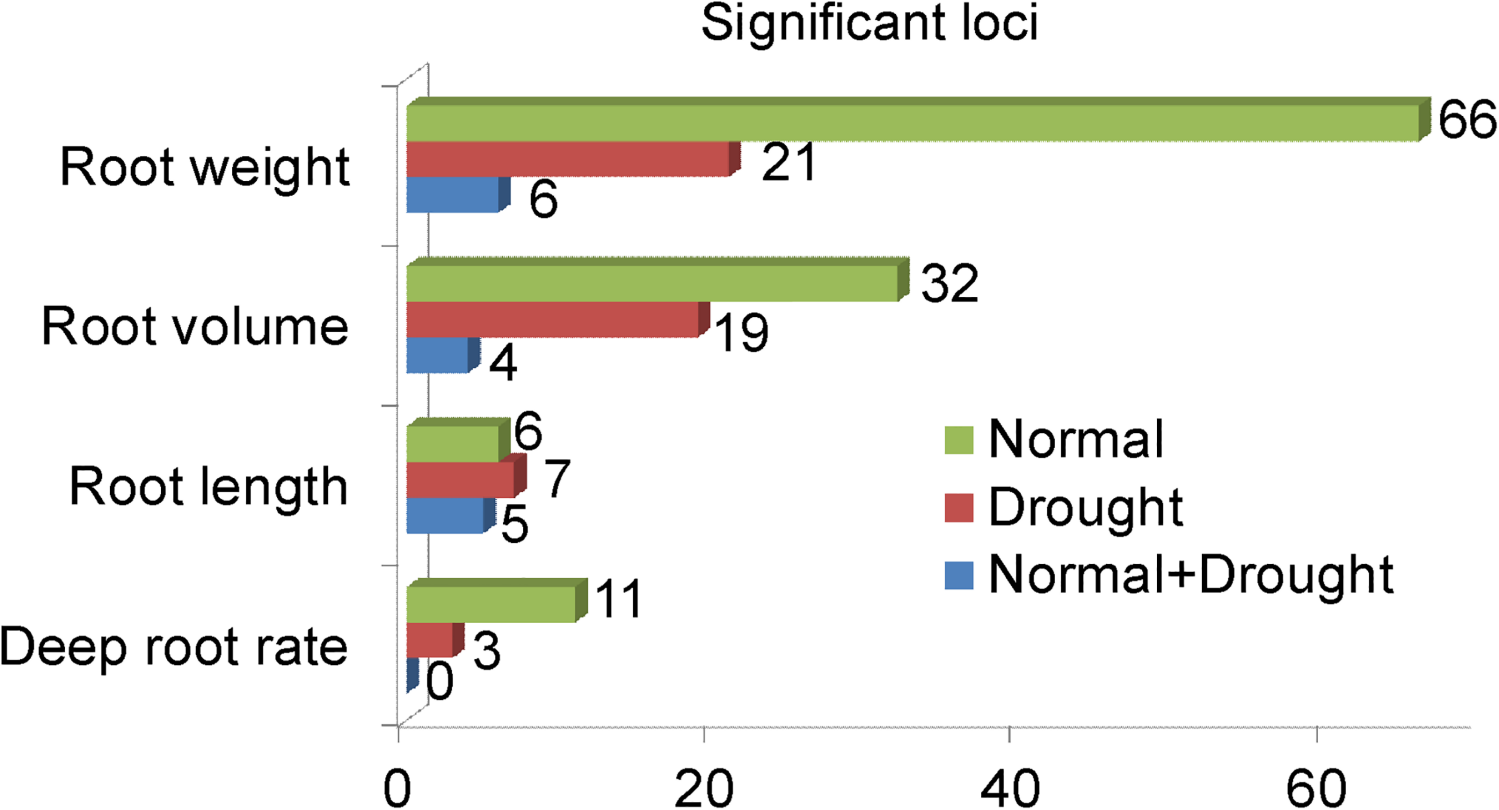
Comparison of significant association loci for four categories of root traits under different conditions. Significant association loci under normal conditions, drought stress conditions, and normal and drought stress conditions simultaneously, are shown in green, red and blue, respectively.

In rice, an association locus has been defined as a chromosomal region in which the distance between the adjacent pairs of associated SNPs is less than 300 kb [27]. According to this definition, a total of 264 suggestive loci containing 373 suggestive SNPs were detected, and 110 of which were significant loci containing 133 significant SNPs (S3 Table). A summary and comparison of the numbers of significant loci for the four categories of root traits under different growth conditions showed that the number of significant loci for root weight was more than that for the other three categories of root traits (Fig 1). In addition, more significant loci were detected under normal conditions than drought stress conditions in all types of root traits, except for root length.

We further checked the loci detected by multiple traits. The result showed that, among the 264 suggestive association loci, a total of 73 loci were detected simultaneously by more than two different traits (S4 Table and S3 Fig). In particular, a locus on chromosome 5, GWAS NO.104, was detected by 8 different traits including RWTN, RVDD, RVTD, RWSN, RWDD, RWTD, RVDN, and RWDN. These results are in agreement with that many of the root traits were highly correlated (S2 Fig).

### Comparison of GWAS results with reported QTLs

**Fig 2:**
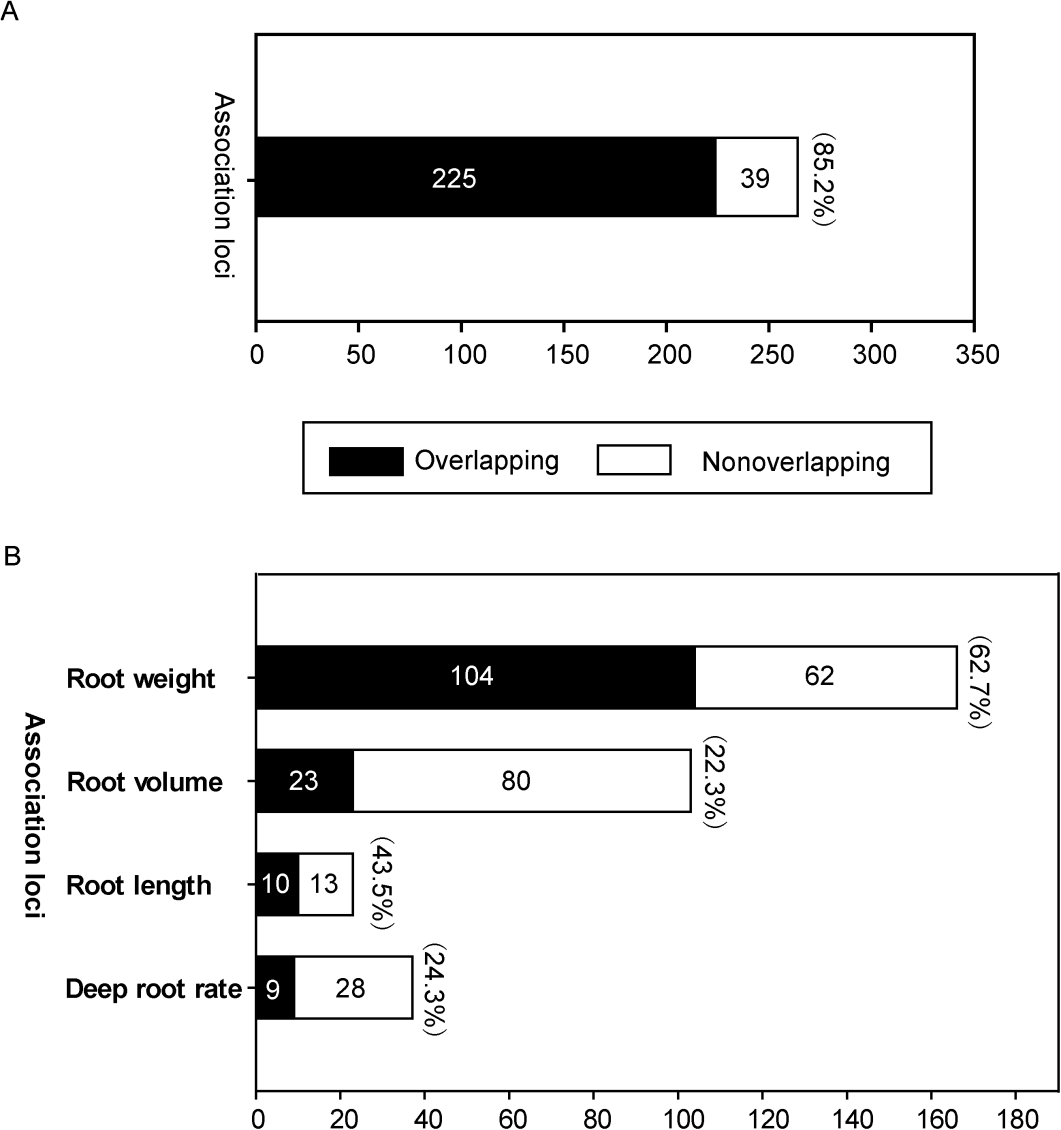
Root association loci overlapping with reported root QTLs. Proportion of root association loci overlapping with reported QTLs for any root-related traits (A) and for the four categories of root traits (B), respectively. The black and white portions of each bar indicate the overlapping loci and nonoverlapping loci, respectively, and the overlapping percentage is shown in bracket at the top of each bar.

In the Tropgene DB (rice data) (http://tropgenedb.cirad.fr/en/rice.html), a large number of QTLs for root traits (deep root rate, root length, root volume, and root weight) in rice have been deposited [31]. We picked up a total of 269 QTLs from this database to compare with the association loci in this study. As the most recent update of the database was in the year 2010, another 36 QTLs [11, 14–16, 18, 19, 32, 33] for the four categories of root traits reported in recent years were also included for comparison (S5 Table). Considering the relevance among deep root rate, root length, root volume, and root weight, we analyzed the overlapping of the association loci and QTLs as a whole firstly, without distinguishing the four types of root traits. The results showed that out of the 264 suggestive loci, 225 loci (85.2%) were located in the region of the QTLs (Fig 2A and S5 Table). The large proportion of association loci overlapping with QTLs suggests that the GWAS is effective in the genetic dissection of root traits in rice.

We further analyzed the overlapping of the association loci and QTLs for the four categories of root traits. The overlapping of the association loci and QTLs for root weight was greater than for the other three types of traits (Fig 2B), with 104 of 166 association loci (62.7%) for root weight locating in the regions of QTLs for root weight (S4 Fig). The detailed information on the overlapping of the association loci and QTLs for root volume (22.3% of the association loci overlapping with the QTLs), root length (43.5% of the association loci overlapping with the QTLs), and deep root rate (24.3% of the association loci overlapping with the QTLs) are presented in S5–S7 Figs, respectively. These results suggest that the significant association loci for root weight related traits may be more repeatable than the loci for the other three types of root traits in the comparison of the two mapping methods.

### Root-related functional genes in the association loci

**Fig 3:**
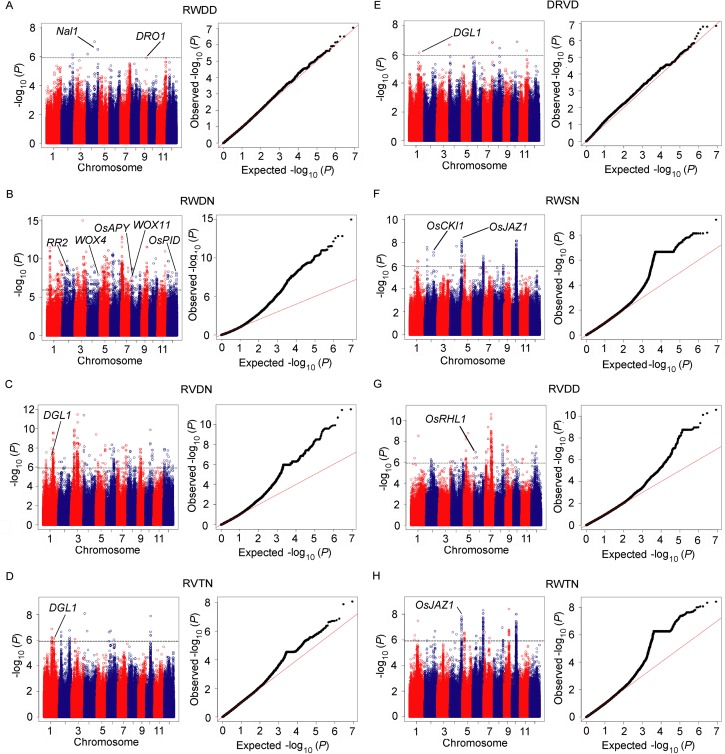
Genome-wide association results for 8 root traits. Manhattan plots (left) and quantile-quantile plots (right) are presented for (A) RWDD, (B) RWDN, (C) RVDN, (D) RVTN, (E) DRVD, (F) RWSN, (G) RVDD, and (H) RWTN. For the Manhattan plots, -log_10_ *P*-values from a genome-wide scan were plotted against the position of the SNPs on each of 12 chromosomes and the horizontal grey dashed line indicates the suggestive threshold (*P* = 1.21×10^−6^). For the quantile-quantile plots, the horizontal axis indicates the -log_10_-transformed expected *P*-values, and the vertical axis indicates the -log_10_-transformed observed *P*-values. The names of reported root-related genes near the association signals are indicated.

Among all of the 264 suggestive loci, 11 reported root related genes were closely linked to the lead SNPs of each suggestive or significant loci for eight different traits (RWDD, RWDN, RVDN, RVTN, DRVD, RWSN, RVDD, and RWTN) (Fig 3 and S6 Table). The details of the GWAS results (Manhattan and quantile-quantile plots) for the other 13 traits are presented in S8 Fig.

The *DEEPER ROOTING 1* (*DRO1*), a gene which increases the deep root ratio by controlling root growth angle [3], was detected in the GWAS NO.198 locus and linked with the trait for RWDD (Fig 3A). This gene is located 14 kb upstream of the lead SNP sf0916321114.

The *WUSCHEL-related homeobox* gene *WOX11*, a gene increasing drought resistance of rice by controlling root hair formation and root system development [34–36], is located in the GWAS NO.161 locus with the lead SNP sf0729023405 for the trait of RWDN (Fig 3B). And within the same locus, *OsAPY*, another gene with a similar genetic effect of controlling root hair formation and root development [37], is located very close to *WOX11* on chromosome 7 (Fig 3B). Also to be mentioned, a cytokinin-responsive gene *RR2*, the common target of *WOX11* and *ERF3* [34], was also associated with the trait of RWDN, in the GWAS NO.43 locus (Fig 3B). Another *WUSCHEL-related homeobox* gene *WOX4* [38], located in the GWAS NO.98 locus, was also associated with RWDN (Fig 3B).

*OsPID*, the ortholog of *PINOID* in rice involved in the control of polar auxin transport and adventitious root development [39], is located in the GWAS NO.263 locus for the trait of RWDN (Fig 3B). *Dwarf and gladius leaf 1* (*DGL1)* [40], a gene involved in crown root development, microtubule organization, and gibberellin signaling, is located in the GWAS NO.19 locus which was simultaneously detected for the traits RVDN, RVTN, and DRVD (Fig 3C–3E). A casein kinase I protein *OsCKI1*, regulating lateral root development in rice [41], is located in the GWAS NO.44 locus associated with RWSN (Fig 3F). *OsRHL1*, a gene controlling root hair formation and development [42], is located in the GWAS NO.120 locus associated with RVDD (Fig 3G).

Among the closely associated candidate genes, there were several reported genes with no evidence for their functions in the control of root traits. Two of them, *Nal1* and *OsJAZ1*, were selected for functional confirmation as case studies.

### Case study 1: *Nal1*, a multi-functional gene controls root volume

*Nal1* has been reported to control leaf width [43], spikelet number [44], photosynthesis rate [45], and yield [46]. And it has also been identified by GWAS for leaf traits [47] and panicle number [48]. In this study, a region including *Nal1* was detected by GWAS for RWDD (dry weight of the deep roots under drought stress conditions), with the *P*-value of 3.12×10^−7^ (Fig 3A), and *Nal1* is located 78-kb downstream of the lead SNP sf0430940007.

**Fig 4:**
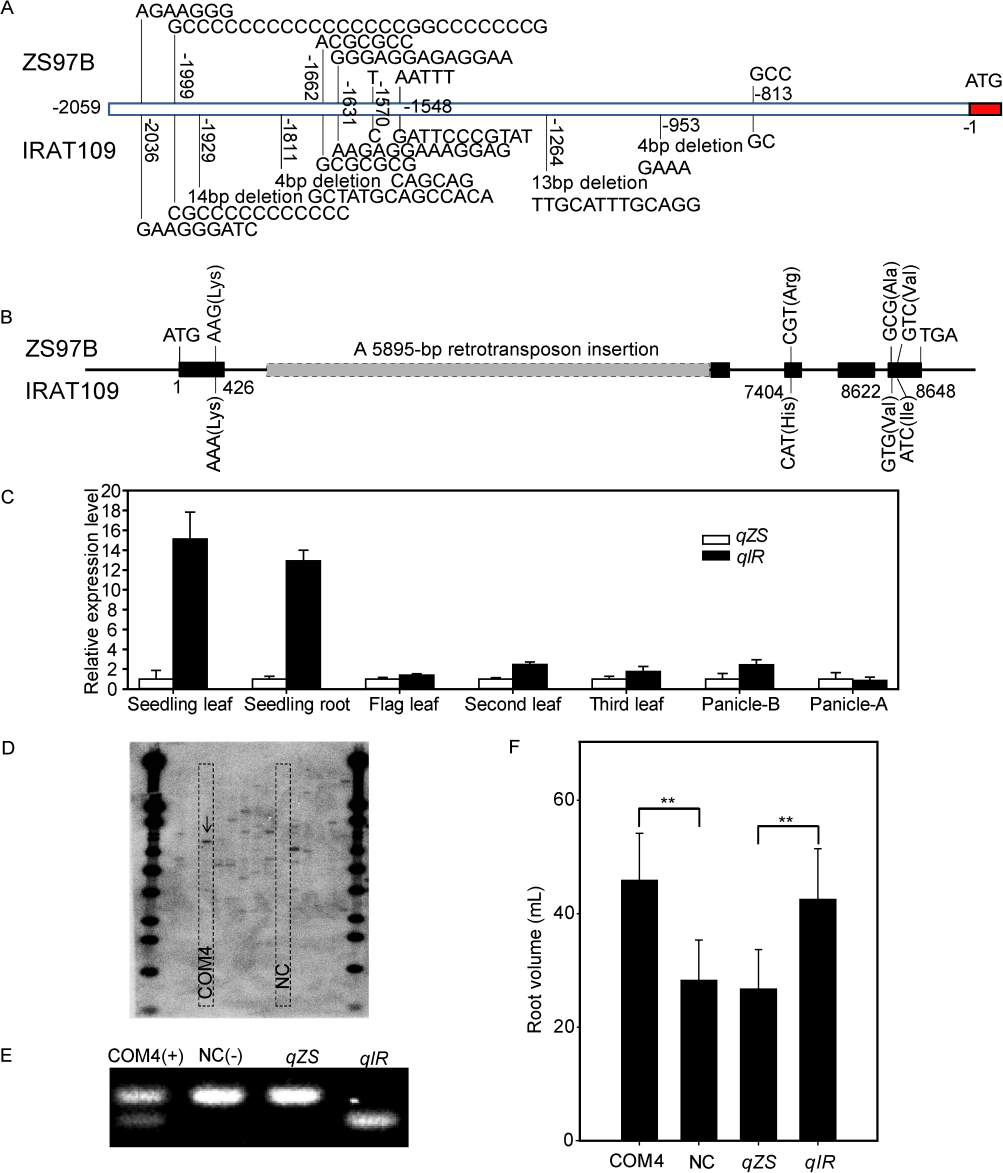
Sequence, expression and phenotypic analyses of *Nal1*. (A) Sequence variations in the promoter (2 kb upstream of the start codon) of *Nal1* between ZS97B and IRAT109. (B) Sequence variations in the ORF of *Nal1* between ZS97B and IRAT109 (the corresponding amino acid changes are shown in parentheses). (C) Relative expression levels of *Nal1* in different tissues of near isogenic lines (NILs) *qZS* and *qIR* at different stages. Panicle-B, panicle before heading; Panicle-A, panicle after heading; (D) Southern blot of complementary transgenic plants. Single copy line COM4 and negative control line NC are indicated by dashed boxes. (E) Genotypic identification of the complementary plants and NILs. (F) Root volume of the complementary lines and NILs. Data represent the mean ± SE (n = 10). **P < 0.01, Student’s *t*-test.

Previously, we fine mapped a pleiotropic QTL *qFSR4* to a region of 38 kb, which controls flag leaf width, spikelet number, and root volume at the reproductive stage in rice. In this region, *Nal1* has been assumed to be the most promising candidate gene [10]. We sequenced the promoter (about 2 kb before the ATG) and CDS region of *Nal1* in the two parent varieties Zhenshan 97B (ZS97B) and IRAT109. Sequence comparison identified one SNP and ten Indels in the promoter region (Fig 4A). In the CDS region, a 5985-bp retrotransposon insertion, located in the junction site of the first intron and the second exon of *Nal1* in the genomes of Koshihikari and Nipponbare [45, 49], was also found in the genomes of ZS97B and IRAT109 (Fig 4B). Except for this insertion, four SNPs were identified in the exons, leading to either synonymous mutations or changes of amino acids with similar biochemical properties, which were unlikely to affect the function of the Nal1 protein (Fig 4B). However, the expression levels of *Nal1* in the near isogenic line (NIL) with a background of IRAT109 (*Nal1*^*IRAT109*^, *qIR*) were significantly greater than in the NIL with the background of ZS97B (*Nal1*^*ZS97B*^, *qZS*) in root, leaf, and panicle (Fig 4C). Therefore, we assumed that the phenotypic difference might be caused mainly by the difference in the expression level of *Nal1*.

To confirm *Nal1* as the causal gene for the pleiotropic QTL *qFSR4* controlling the root traits, we performed a genetic complementation analysis. The 1,749-bp full-length CDS of *Nal1* from the NIL of *qIR*, driven by the 2,059-bp promoter from the NIL of *qIR*, was transformed into the NIL of *qZS*. The positive complementary line COM4 with single copy of the transgene (Fig 4D) showed significantly greater root volume (Fig 4F), wider flag leaves, and bigger spikelet number (S9 Fig) compared to the negative transgenic control (NC). This result suggests that *Nal1* is the causal gene of the pleiotropic QTL *qFSR4*.

**Fig 5:**
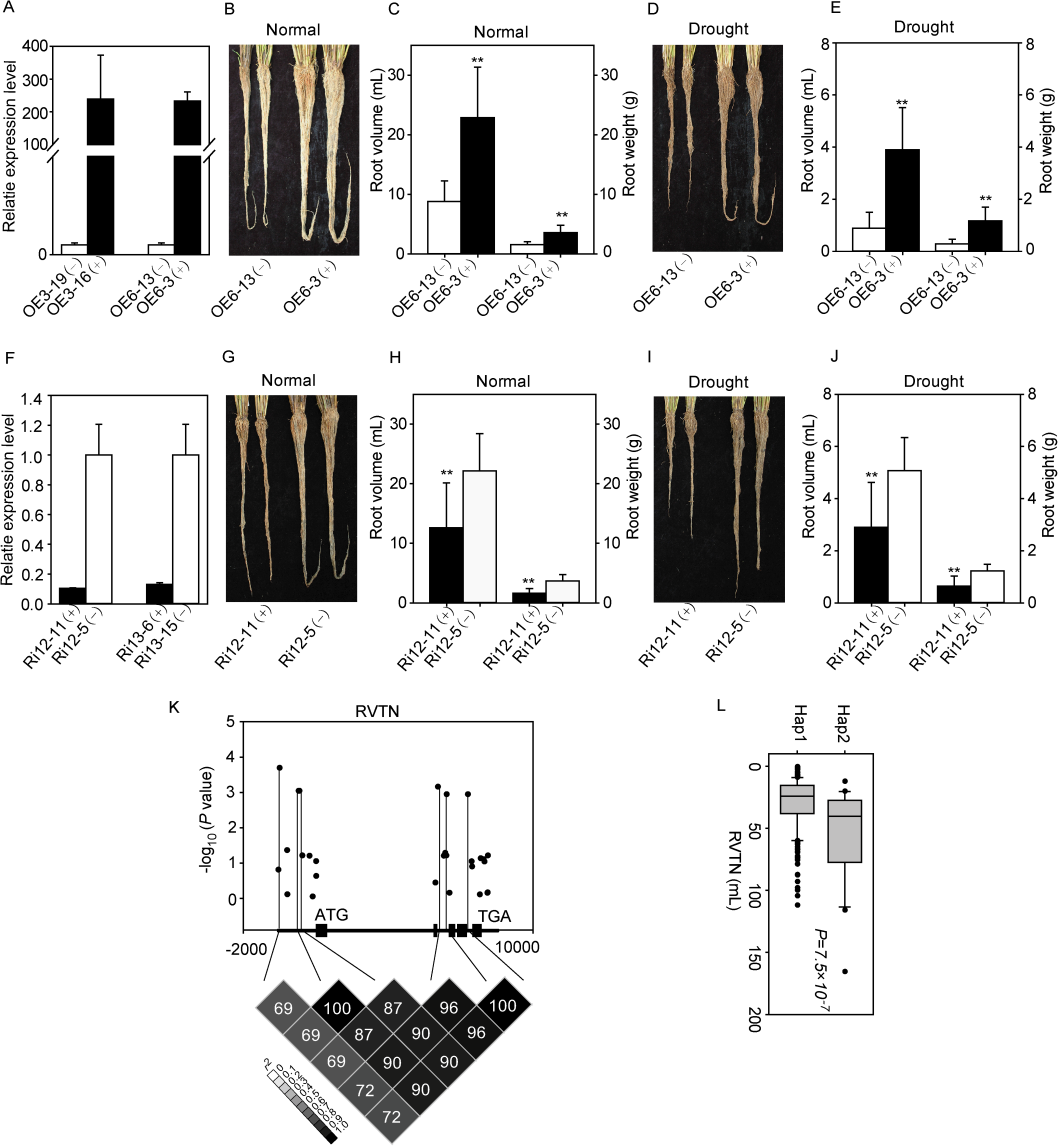
Root phenotypes of *Nal1* transgenic plants and association analysis of *Nal1*. (A, F) Relative expression level of *Nal1* in two overexpression lines (A) and two RNAi lines (F), compared to the corresponding segregated negative control under normal conditions. (B, D) Visual root phenotypes of the *Nal1*-overexpression (OE6-3(+)) plants and the segregated negative-transgenic control (OE6-13(-)) at the seed maturation stage in PVC tubes under normal (B) and drought stress (D) conditions. (C, E) Root volume and weight of OE6-3(+) plants and OE6-13(-) control at the seed maturation stage in PVC tubes under normal (C) and drought stress (E) conditions. Data represent the mean ± SE (n = 10). **P < 0.01, Student’s *t*-test. (G, I) Visual root phenotype of the *Nal1*-RNAi (Ri12-11(+)) plants and the segregated negative-transgenic control (Ri12-5(-)) at the seed maturation stage in PVC tubes under normal (G) and drought stress (I) conditions. (H, J) Root volume and weight of Ri12-11(+) plants and Ri12-5(-) at the seed maturation stage in PVC tubes under normal (H) and drought stress (J) conditions. Data represent the mean ± SE (n = 10). **P < 0.01, Student’s *t*-test. (K) Association analysis of sequence variations of *Nal1* with RVTN and the pattern of pair wise LD of associated SNPs. (L) RVTN of two main haplotypes of *Nal1*.

To further confirm whether the variation in the expression level of *Nal1* leads to the phenotypic difference, we constructed an overexpression vector and an RNA interference vector using the cDNA of *Nal1* from the rice variety Nipponbare. Two independent overexpression lines and RNA interference lines of *Nal1* were used for root traits investigation under normal and drought stress conditions. The expression levels of *Nal1* in these lines under normal conditions at the seedling stage were presented in Fig 5A and 5F. Compared with the segregated negative transgenic control plants (OE6-13(-)), overexpression plants (OE6-3(+)) exhibited a larger root size (Fig 5B), with significantly increased root volume and root weight under normal growth conditions at the seed maturation stage (Fig 5C), and the difference was also observed under drought stress conditions (Fig 5D and 5E). To the contrary, RNA-interference plants (Ri12-11(+)) exhibited significantly smaller root volume and root weight under both normal (Fig 5G and 5H) and drought stress (Fig 5I and 5J) conditions, compared to the segregated negative transgenic control plants (Ri12-5(-)). The same difference in root phenotypes was observed for another overexpression line (OE3) and another RNA interference line (Ri13) (S10 Fig). These results further confirmed that *Nal1* is the causal gene for the QTL *qFSR4* controlling root volume.

Since *Nal1* was identified to control root volume, we examined the association of sequence variation in *Nal1* with the root volume trait RVTN (Fig 5K). In the 2 kb promoter region and the entire coding region, a total of 78 SNPs were extracted from RiceVarMap database [50], and 28 SNPs were used for association analysis after excluding minor variants (frequency < 0.05) (S7 Table). Among them, six SNPs were associated with the RVTN, surpassing the Bonferroni-adjusted *P*-value (0.05/total markers = 1.78×10^−3^) in a mixed linear model. Except for three associated SNPs in the promoter, the other three SNPs were in the intron or 3' UTR region. Haplotype analysis of the six associated SNPs revealed two major haplotypes (S7 Table), and Hap2 had a greater RVTN than Hap1, with a *P-*value of 7.5×10^−7^ (Fig 5L). Considering the difference in the expression level of *Nal1* observed in the *qIR* and *qZS* NILs, this result implies that sequence variations in the promoter region of *Nal1* may mainly contribute to the phenotypic difference in RVTN.

### Case study 2: A reported gene *OsJAZ1* has novel function associated with root traits

A locus with a lead SNP sf0433137256 on chromosome 4 was identified for RWSN (dry weight of the shallow roots under normal conditions) with a *P*-value of 6.4×10^−9^ (Fig 3F) and for RWTN (dry weight of the total roots under normal conditions) with a *P*-value of 1.58×10^-8^ (Fig 3H). A reported gene *OsJAZ1 (EG2)*, which has a role in the regulation of spikelet development in rice [51], is located only 14 kb upstream of the lead SNP. This gene caught our attention because it has a strong expression level in root [52], and it is one of the few genes with an obvious root-enriched expression pattern in the region around the lead SNP according to data from RiceXPro database (http://ricexpro.dna.affrc.go.jp/) [53] (S11 Fig).

**Fig 6:**
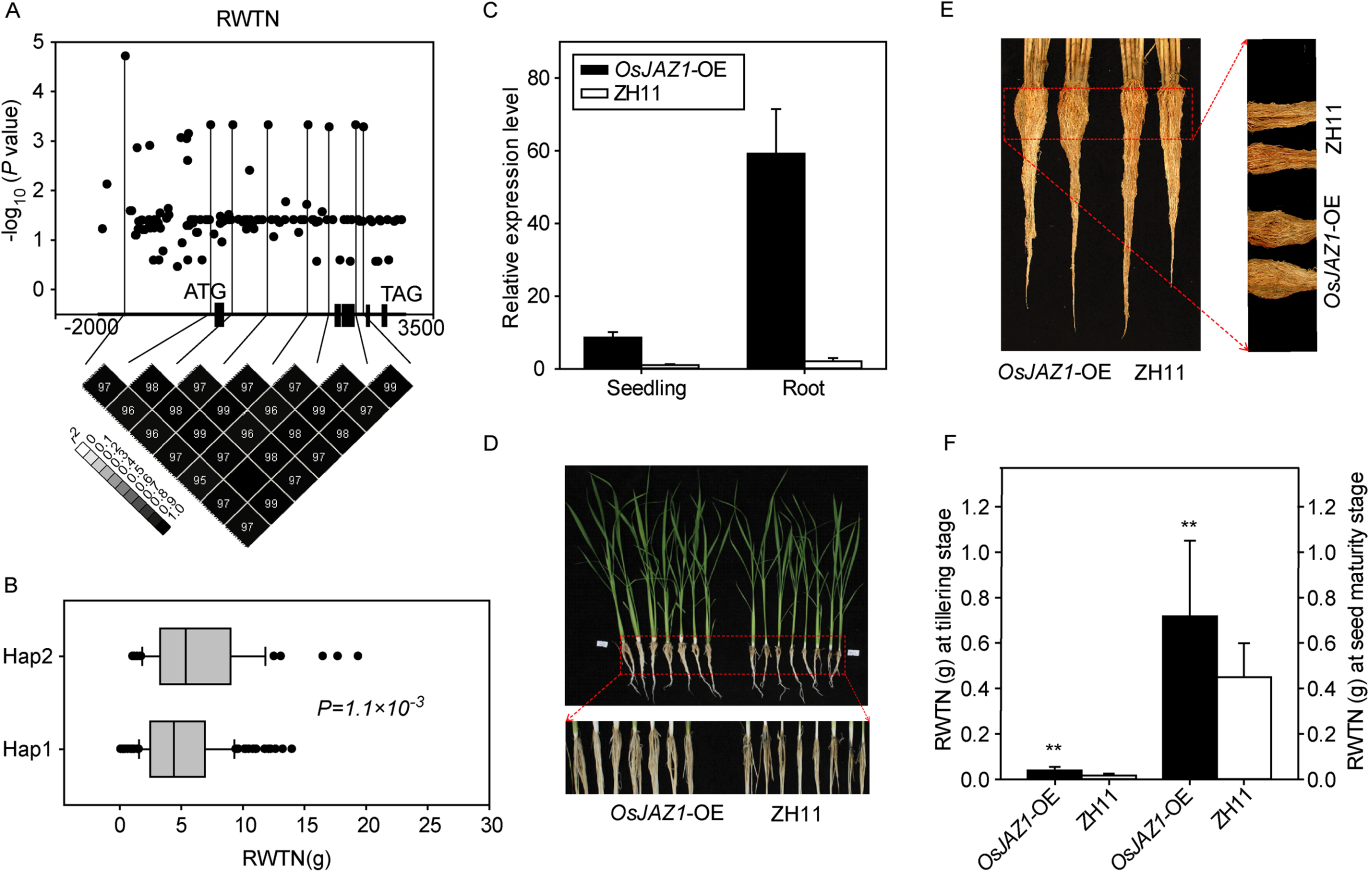
Association analysis of *OsJAZ1* and the root phenotypes of *OsJAZ1*-OE plants. (A) Association analysis of the genetic variation of *OsJAZ1* with RWTN and the pattern of pair wise LD of the associated SNPs in *OsJAZ1*. (B) RWTN of two main haplotypes of *OsJAZ1*. (C) Relative expression levels of *OsJAZ1* in the seedling and root of *OsJAZ1*-OE plants and wild-type ZH11 at the seedling stage. (D, E) Visual root phenotypes of OE and ZH11 plants at the tillering (D) and the seed maturation (E) stages. (F) Root dry weight of OE and ZH11 plants at the tillering and the seed maturation stages. Data represent the mean ± SE (n = 15). **P < 0.01, Student’s *t*-test.

Association analysis between *OsJAZ1* and RWTN was conducted to detect the causal variant. A total of 143 SNPs in the 2kb promoter region and the entire coding region of *OsJAZ1* from RiceVarMap database were used for study after excluding minor variants (frequency < 0.05) (S8 Table). It was shown that only one SNP (*P* = 1.94×10^−5^), located in the promoter region, surpassed the Bonferroni-adjusted *P*-value (0.05/total markers = 3.49×10^−4^) in a mixed linear model, and it was regarded as a suggestive association with the RWTN (Fig 6A). Meanwhile, seven SNPs in the promoter region and intron region were identified as marginally suggestive. Further haplotype analysis of the above eight associated SNPs revealed two major haplotypes (S8 Table), and Hap2 had significantly greater RWTN value than Hap1 (*P* = 1.1×10^−3^) (Fig 6B).

To examine whether *OsJAZ1* is functionally related to root traits, we checked the phenotype of *OsJAZ1*-overexpression (*OsJAZ1-*OE) plants. The *OsJAZ1-*OE plants exhibited significantly longer roots and more crown roots than the wild-type Zhonghua11 (ZH11) at the seedling stage (S12A and S12B Fig). The relative expression level of *OsJAZ1* in the leaves of *OsJAZ1-*OE plants was about ten times greater than in ZH11, while the difference in the roots was about thirty times (Fig 6C). The *OsJAZ1-*OE plants also showed significantly larger root size at the tillering stage (Fig 6D) and the seed maturation stage (Fig 6E). The average root dry weight of the *OsJAZ1-*OE plants was significantly greater than that of ZH11 at these two stages (Fig 6F), which is consistent with the association result that the locus containing *OsJAZ1* was detected for the trait of root weight. These results suggest that *OsJAZ1* may regulate root development at various developmental stages of rice.

## Discussion

To the best of our knowledge, this is the first attempt to conduct a genome-wide association study for root traits of rice growing in soil under normal and drought stress conditions at the reproductive stage. In this study, 264 suggestive association loci for root traits under normal and/or drought stress conditions were identified, and 11 reported root-related genes, including *DRO1*, *WOX11*, and *OsPID*, were found to be located in these loci. A total of 225 suggestive loci are overlapped with the reported root related QTLs, suggesting the excellent reliability of the genetic loci of root traits identified in this study. More importantly, the case studies of *Nal1* and *OsJAZ1* demonstrated that the association loci detected in this study are highly valuable for further identification of the causal genes for root traits, and some of these loci may be the promising candidates for genetic improvement of root architecture and drought resistance.

### Root phenotyping of plants growing in soil

Plant roots are of great significance in drought avoidance. However, phenotyping of the root system in soil is still a big challenge, especially for plants under drought stress conditions. *DRO1* [14], *DRO2* [15], and *DRO3* [16], three major QTLs involved in the deep rooting of rice under normal conditions, have been fine mapped by planting rice in hemispherical baskets placed in hydroponic solution. The hemispherical basket method was adopted in the GWAS for deep root ratio analysis in the field [24]. Association mapping of root traits in a *japonica* rice panel has also been conducted using a hydroponic root phenotyping system with glass beads to support the plants [23], but root phenotyping under drought stress conditions was seldom addressed. Although the hydroponic method can overcome the problem of root invisibility which exists with the soil method, natural root architecture can hardly be revealed [54]. In this study, root traits of rice under normal and drought conditions were measured using the PVC tube method [9]. The PVC tube, 1 m in height and 20 cm in diameter, was designed specifically for measuring the root traits of rice under normal or drought stress conditions. Using this method, we could measure several important root traits that are closely related to drought avoidance in the field, such as the maximal root length, root dry weight, root volume, and deep root rate. Despite the labor-intensive root washing, root phenotypes of plants grown in soil-filled large pots are more similar to the actual root system in the field than the hydroponic method.

Nevertheless, the PVC tube method also has its disadvantages in root phenotyping. First, it is hard to monitor the dynamic growth of root traits unless a more powerful detection system is developed. Second, sandy soil should be carefully washed away from the roots before measuring, which inevitably impairs the natural root architecture. Therefore, a non-destructive root phenotyping system is eagerly expected. Nowadays, several research groups have successfully established different kinds of non-destructive root phenotyping systems such as RooTrak system [54]. However, a high throughput root phenotyping technology for plants grown in natural soil conditions remains a big challenge.

### Difference in genetic control of root traits under normal and drought stress conditions

Some root traits showed differences under normal conditions and drought stress conditions. Due to the effect of population structure, the change tendency of deep root rate traits after drought stress treatment varied not only between the *indica* and the *japonica *subpopulations but also in the different *indica* subpopulations (*ind*, *ind I*, and *ind II*) and the different *japonica* subpopulations (*jap*, *tej*, and *trj*) (Table 2). Comparison of the association loci under normal and drought stress conditions suggests that some loci can be detected under both normal and drought stress conditions. The genetic control for some traits under normal and drought stress conditions is partially overlapped. For example, for RVTN and RVTD, two very close associations were detected on chromosome 4 (Figs 3D and S8I), and these two associations were regarded as the same association locus (GWAS NO.78). Some loci were detected only under normal conditions or drought stress conditions. These results suggest that the genetic basis of root traits under normal and drought stress conditions is largely different for some traits, such as the maximal root length, root volume, and deep root weight. For an example, several obvious peaks were detected for deep root weight under normal conditions (RWDN) (Fig 3B), but few signals were detected for the trait under drought stress conditions (RWDD) (Fig 3A). It is noteworthy that a significant locus (GWAS NO.198) was detected on chromosome 9 with a lead SNP (sf0916321114). However, this locus was not detected under normal conditions for RWDN. Interestingly, *DRO1* is located at 14 kb upstream of the lead SNP. *DRO1* has been reported to be involved in cell elongation of the root tip in response to gravity, and greater expression of *DRO1* could increase deep rooting by increasing the root growth angle, thus improving drought avoidance and maintaining yield under drought stress conditions. The single 1-bp deletion in the fourth exon of *DRO1* in IR64 results in the introduction of a premature stop codon [3]. In the 529 accessions used in this study, however, only two accessions have the 1-bp deletion (S9 Table). Therefore, this 1-bp deletion is unlikely to be the causal variant for the root traits in this panel.

### The complexity of genetic control of root traits and exploration of candidate genes

Compared to many dicot species such as *Arabidopsis*, which has a primary root iteratively branching to generate several orders of lateral roots, the root system of cereal crops such as rice is more complex [55]. The complexity of the genetic control of root architecture is as complex as the trait itself [4, 9]. In this study, 73 loci were detected for two and more traits (S4 Table), but 191 loci were detected only for one trait, which may suggest that some loci have pleiotropic effects on root traits while most of the root traits are controlled by distinct genetic loci. On the other hand, considering the relatively low LD decay in rice, one association locus in this study was defined as a 200 kb region containing more than ten genes, thus it is rather difficult to pinpoint the causal genes for these loci. However, the combination of QTL information, expression profile, and prediction of gene function could help to narrow down the candidate genes, just like the two case studies we presented.

The pleiotropic QTL *qFSR4* affects flag leaf width, spikelet number, and root volume in rice, and it has been fine mapped to a 38 kb region in which *Nal1* was assumed to be the most likely candidate gene [10]. *Nal1* has been reported to be involved in auxin polar transport [43] and cell division [56], and its regulation in the development of leaves and adventitious roots may be via modulating the expression of the *PIN* and *CRL* genes [57]. In this study, the association analysis revealed a locus for RWDD, which overlapped with the QTL *qFSR4*. We confirmed the function of *Nal1* in the control of root traits by sequence and expression level comparison, genetic complementation, and haplotype analysis. Overexpression and RNA-interference lines of *Nal1* resulted in significant changes in root volume and root weight under both normal and drought stress conditions compared to the control plants, demonstrating the authentic function of *Nal1* in the control of root traits. The *Nal1* allele from the *japonica* rice cultivar showed better performance in regulating adventitious root development at the seedling stage [57], which is consistent with our result that the *japonica* allele of *Nal1* is superior when compared to the *indica* allele with respect to the control of root volume at the adult stage.

Another case study for *OsJAZ1*, a candidate causal gene for the locus detected by RWSN and RWTN on chromosome 4, suggests that the association results could provide opportunities to identify novel genes or known genes with new functions in the control of root traits. *OsJAZ1* has been reported to be involved in spikelet development in rice [51]. However, no JAZ proteins have been reported to regulate root traits under normal or drought stress conditions in rice, although some JAZ proteins in *Arabidopsis* have been shown to regulate root development under JA treatment [58]. Here, we found *OsJAZ1* was involved in root development with genetic evidence of *OsJAZ1*-overexpression lines at the seedling, tillering and reproductive stages. It would be very interesting to further reveal how *OsJAZ1* regulates both the underground organ (root) and the reproductive organ (spikelet).

Besides the tens of reported root-related QTLs or genes, many unreported loci or genes for root traits were detected in our association analysis. With the help of expression profiling databases and bioinformatic analysis, we could narrow down the potential candidate causal genes for root traits in the significant association loci. For example, 28 possible candidate genes with obvious drought-responsive and/or root-specific expression patterns were identified for some of the significant association loci (S10 Table). Nevertheless, subsequent genetic experiments are necessary to confirm the functions of these genes in root development or drought avoidance.

In conclusion, our study provides a relatively comprehensive analysis of the genetic architecture of root traits in rice. We employed a GWAS with 529 rice accessions for root traits at the seed maturation stage under normal and drought stress conditions, and 225 of 264 loci identified by GWAS overlapped with reported root related QTLs. Many known root-related genes were located in the significant association loci. Importantly, case studies of two genes, *Nal1* and *OsJAZ1*, demonstrate the feasibility of mining for candidate genes by GWAS. The association loci and causal genes identified in this study provide an important foundation for revealing the molecular mechanism of root development and genetic improvement of rice root or drought resistance in the future.

## Materials and methods

### Population materials, growth conditions, and drought stress treatment

A total of 529 rice accessions including 202 from the China Core Collection and 327 from the World Core Collection were used for the association analysis (S1 Table). This panel of rice accessions is essentially the same as the panel of 533 accessions as previously described [29], except three accessions (C126, W196, and W232) with severe heterozygosity and one (W190) with a low mapping rate (10%) omitted.

For GWAS of root traits, 529 rice accessions were grown in PVC tubes (1 m in height, 20 cm in diameter) described by Yue et al [9], with one plant per tube and six plants per accession (three for normal conditions and three for drought stress conditions). The PVC tubes were arranged in the field facilitated with a moveable shelter at the experimental station of Huazhong Agricultural University (114.33°E, 30.35°N). The average air temperature was 30.3°C and the average relative air humidity was 67.5% during the rice growth period. The arrangement of the PVC tubes and rice accessions are shown in S13A and S13B Fig. Considering that the heading date varied in the whole population, the 529 accessions were sown in several batches each with a relatively close heading date. At the beginning of the tillering stage, 1 g of urea (dissolved in water) was applied to each tube. The plants were fully irrigated every day until the drought stress treatment was applied. At the booting stage, drought stress was applied to three of the blocks with the other three blocks used as a control. To apply drought stress treatment, water was added to the full capacity of the tubes, and the plugs on the tubes were removed, allowing the slow drainage of water in the tubes through the small holes on the tubes. Rain was kept off by closing the shelter. When all the leaves of a stressed plant became fully rolled, watering was applied to the full capacity of the tubes. When the full water capacity maintained for one day, the second cycle of drought stress was applied to the plant until all of the leaves became fully rolled again. After the second round of drought stress treatment, watering was resumed for the rest of the life cycle.

### Root phenotyping procedure of 529 rice accessions

A total of 21 root related traits were phenotyped in this study. The root traits were measured at the seed maturation stage of the plants. To measure these traits, the plastic bag containing the soil and roots was pulled out from the PVC tube and laid out on a soil-washing table with a 2-mm sieve. After removing the plastic bag, the soil was washed away carefully and the length of the longest root was scored as the maximum root length (in centimeters). The washed roots which were free from the soil were shown in S13C Fig. Then the roots were cut into two parts at 30 cm from the basal node of the plant. The volume (in milliliters) of the roots from the two parts was measured in a cylinder using the water-replacement method. The dry weight (in grams) of the roots from the two parts was measured with a balance after air-drying the roots. The root mass above 30 cm was designated as shallow roots while the root mass below 30 cm was designated as deep roots, from which a number of indices were derived. The flow of measurement is shown in S13D Fig. The abbreviations and descriptions of the corresponding traits are listed in Table 1.

### Genome-wide association study

A total of 529 accessions including nine subpopulations were collected to construct this association panel. For the 21 traits used for GWAS, we adopted a mixed-model approach using the factored spectrally transformed linear mixed models (FaST-LMM) program, with 4,358,600 SNP across the entire rice genome (minor allele frequency ≥ 0.05; the number of accessions with minor alleles ≥ 6). The suggestive and significant *P*-value thresholds of the entire population were respectively 1.21×10^−06^ and 6.03×10^−08^. The linkage disequilibrium (LD) statistic *r*^2^ was calculated by Plink based on haplotype frequencies. More detailed information about our association analysis was referenced in the recent study [29]. Candidate association analysis of *Nal1* and *OsJAZ1* was performed with TASSEL version 5 [59]. LD plots were generated with Haploview4.2, and LD was indicated using *r*^2^ values between the pairs of SNPs multiplied by 100 (white, *r*^2^ = 0; shades of gray, 0< *r*^2^ <1; black, *r*^2^ = 1).

### Overlapping analysis of QTLs and association loci

The physical region of root-related QTLs was determined by the physical position of the left border marker and the right border marker, which were obtained by searching markers information in the GRAMENE database (http://www.gramene.org/). The physical region of each association locus was defined as 200 kb around each lead SNP. An overlapping locus was claimed if the physical region of the association locus is overlapped with the physical region of any reported QTL for root traits.

### Haplotype analysis

The genotypes of *Nal1* and *OsJAZ1* in the 529 rice samples were obtained from the RiceVarMap database (http://ricevarmap.ncpgr.cn/). The haplotypes were classified based on all of the SNPs with an MAF > 0.05 in a candidate gene. The haplotypes containing at least ten rice accessions were used for comparative analysis. One-way ANOVA and Student’s *t*-test was employed to compare the differences in root traits among the haplotypes [60].

### Generation and identification of transgenic materials

The QTL *qFSR4* was narrowed down to a region of approximately 38-kb flanked by markers FSR-75 and FSR-78 [10], and the progeny of a recombinant plant WHD10-74 were genotyped with the marker RM17483, and the plant with homozygous genotype as ZS97B was used as NIL *Nal1*^ZS97B^ (*qZS*) while the plant with homozygous genotype as IRAT109 was used as NIL *Nal1*^IRAT109^ (*qIR*) in our study. For the complementation test of *Nal1*, a 2,059-bp *Nal1* promoter fragment was amplified from the NIL *qIR* with the addition of restriction sites for *Kpn*I and *Eco*RI, and the 1,749-bp *Nal1* full CDS fragment was amplified from the cDNA of the *qIR* line with the addition of a restriction site for *Kpn*I at both ends. The two fragments were cloned into the binary vector pCAMBIA2301 to generate the transformation plasmid for the complementation test. The resulting transformation plasmid was introduced into NIL *qZS* by *Agrobacterium*-mediated transformation [61]. The copy numbers of marker gene (G418) for transformation were determined by Southern blot. The genotype was detected with PCR using the primer pair based on the sequence difference in the promoter region of ZS97B and IRAT109.

For the *Nal1* overexpression transgenic plants, the full-length cDNA of *Nal1* was amplified from the *japonica* cv. Nipponbare. The sequence-confirmed PCR fragment was ligated into pCAMBIA1301U which was digested with *Kpn*I, based on the Gibson assembly principle [62]. The construct was introduced into the *japonica* cv. Zhonghua11 by *Agrobacterium*-mediated transformation [61].

For the *OsJAZ1* overexpression transgenic plants, the full-length cDNA of *OsJAZ1* was cut from a clone from the full length cDNA library of the *indica* cv. Minghui63, and cloned into the pCAMBIA1301H vector driven by the *OsLEA3-1* promoter *LEAP* [63]. The construct was introduced into the *japonica* cv. Zhonghua11 by *Agrobacterium*-mediated transformation [61].

For the *Nal1* interference transgenic plants, the 454-bp length cDNA of *Nal1* was amplified from the *japonica* cv. Nipponbare by RT-PCR. The sequence-confirmed PCR fragment was recombined into the pANDA vector [64], by the gateway system. The construct was introduced into *japonica* cv. Zhonghua11 by *Agrobacterium*-mediated transformation [61].

### Phenotyping NILs and transgenic plants for candidate genes

To investigate the transcript levels of *Nal1* in different tissues at different stages, seeds of the NILs *qZS* and *qIR* were germinated on normal 1/2 strength MS medium in Petri dishes. Before germination, rice seeds were surface-sterilized in 75% ethanol for 5 min, followed by a 10 min incubation with 0.15% HgCl_2_, and then washed four to five times with sterile water. After germination, the seedlings were transplanted to 10 cm high sterile plastic box with a 5 cm deep normal 1/2 strength MS medium in it. The roots and leaves of *qZS* and *qIR* seedlings were sampled at the two-leaf stage, then the *qZS* and *qIR* seedlings were transplanted in the field. Flag leaves, the second leaves, and the third leaves of *qZS* and *qIR* were sampled at the heading stage. Panicles of *qZS* and *qIR* were sampled before heading and after heading.

To investigate the transcript levels of *OsJAZ1* and the root phenotype of *OsJAZ1* overexpression and wild-type plants Zhonghua11 (ZH11), the positive transgenic plants were selected by germinating on 1/2 strength MS medium containing 25 mg/L hygromycin B (Roche). The wild-type seeds were germinated on normal 1/2 strength MS medium. Before germination, the rice seeds were surface-sterilized in 75% ethanol for 5 min, followed by a 10 min incubation with 0.15% HgCl_2_, and then washed four to five times with sterile water. After germination, the overexpression seedlings were transplanted to 10 cm × 10 cm square plastic Petri dishes with a 0.5 cm deep normal 1/2 strength MS medium in it (14 seedlings each, three repeats), with control plants in the same dish (half of each). Root length, seedling length, and crown root number of the overexpression and wild-type plants were investigated at the two-leaf stage. The roots and leaves of overexpression and wild-type plants were sampled after phenotype investigation. The seedlings were transplanted to pots with sandy soil in it until the tillering stage, at which point the seedlings were carefully washed out from the sandy soil and the roots were cut for root weight measurement after air drying. Then, the seedlings were transplanted to PVC tubes (each with one overexpression plant and one wild-type plant), and the root phenotype of the overexpression and wild-type plants were investigated at the seed maturation stage using the same method as mentioned above.

To investigate the phenotype of the NILs of *qFSR4* and complementary plants, the seeds of *qZS*, *qIR*, COM4, and NC were sown in the nursery field and the seedlings were transplanted to PVC tubes at the four-leaf stage. Each tube contained two plants, one *qZS* plant and one *qIR* plant, or one COM4 plant and one NC plant. Each group was repeated ten times (tubes) for normal growth and drought stress treatment, respectively. Leaf width was measured for three flag leaves of each plant at the heading stage, and the panicles were harvested for counting spikelet and seed numbers after maturation. The root traits were measured by carefully washing soil away using the method described above.

To investigate the root phenotype of the *Nal1* overexpression and RNA-interference plants, the seeds of transgenic positive plants (OE3-16(+), OE6-3(+), Ri12-11(+), and Ri13-6(+)) and negative controls (OE3-19(-), OE6-13(-), Ri12-5(-), and Ri13-15(-)) were sown in the nursery field, leaves of each line were sampled at the four-leaf stage for investigating the transcript levels of *Nal1*. Then the seedlings were transplanted to PVC tubes. Each tube contained two plants (overexpression or RNAi plant and corresponding control). Each group was repeated ten times (tubes) for normal growth and drought stress treatment, respectively. Drought stress treatment was applied at the booting stage as described above. The traits of root volume and root weight were investigated as described above.

### RNA extraction and expression analysis

Total RNA was extracted using Trizol reagent (Invitrogen). The first-strand cDNA was reverse transcribed using M-MLV reverse transcriptase (Invitrogen) according to the manufacturer’s instructions. Quantitative PCR was conducted on a 7500 Real-Time PCR System (Applied Biosystems) using SYBR Premix ExTaq (TaKaRa) according to the manufacturer’s instructions. The rice *Ubiquitin* gene was used as the internal control. The relative expression level was determined as reported previously [65].

## Supporting information

S1 Fig Distribution of 21 root traits. (TIF)

S2 Fig Correlation coefficient among 21 root traits. The correlation coefficients are shown in the lower left part of the figure. The correlation coefficients are indicated by the color (referring to the scale on the right) and size of the circles in the top right part of the figure.
(TIF)

S3 Fig Co-localization of significant associations among different traits. The vertical long bars represent chromosomes and black dots represent centromeres. Scale bar, 5 million base pair.
(TIF)

S4 Fig Overlaps between the association loci with the QTL for root weight. The vertical long bars represent chromosomes, the short black bars represent the QTLs for root weight, and the asterisks represent the association loci for root weight. The numbers corresponding to asterisks represent the lead SNPs for the association loci. Scale bar, 5 million base pair.
(TIF)

S5 Fig Overlapping of the association loci with the QTL for root volume. The vertical long bars represent chromosomes, the short black bars represent the QTLs for root volume, and the asterisks represent the association loci for root volume. The numbers corresponding to the asterisks represent the lead SNPs for the association loci. Scale bar, 5 million base pair.
(TIF)

S6 Fig Overlapping of the association loci with the QTL for root length. The vertical long bars represent chromosomes, the short black bars represent the QTLs for root length, and the asterisks represent the association loci for root length. The numbers corresponding to the asterisks represent the lead SNPs for the association loci. Scale bar, 5 million base pair.
(TIF)

S7 Fig Overlapping of the association loci with the QTL for deep root rate. The vertical long bars represent chromosomes, the short black bars represent the QTLs for deep root rate, and the asterisks represent the association loci for deep root rate. The numbers corresponding to the asterisks represent the lead SNPs for the association loci. Scale bar, 5 million base pair.
(TIF)

S8 Fig Genome-wide association results of 13 root traits. Manhattan plots (left) and quantile-quantile plots (right) are presented for (A) MRLN, (B) MRLD, (C) MRLR, (D) DRLN, (E) DRLD, (F) RVSN, (G) DVRN, (H) RVSD, (I) RVTD, (J) RWSD, (K) DWRN, (L) RWTD, and (M) DWRD. For the Manhattan plots, -log_10_ *P*-values from a genome-wide scan were plotted against the position of the SNPs on each of 12 chromosomes, and the horizontal grey dashed line indicates the suggestive threshold (*P* = 1.21×10^−6^). For the quantile-quantile plots, the horizontal axis indicates the -log_10_-transformed expected *P*-values, and the vertical axis indicates the -log_10_-transformed observed *P*-values.
(TIF)

S9 Fig Leaf and panicle phenotype of the complementary lines and NILs. Flag leaf width (A) and spikelet number per panicle (B) of the complementary lines and NILs. The data represent the mean ± SE (n = 10). **P < 0.01, Student’s *t*-test.
(TIF)

S10 Fig Root phenotype of the *Nal1*-OE3 line and *Nal1*-Ri13 line under normal and drought stress conditions. (A, B) Visual root phenotypes of the *Nal1*-overexpression (OE3-16(+)) plants and the segregated negative-transgenic control (OE3-19(-)) at the seed maturation stage in PVC tubes under normal (A) and drought stress (B) conditions. (C, D) Visual root phenotypes of the *Nal1-*RNAi (Ri13-6(+)) plants and the segregated negative-transgenic control (Ri13-15(-)) at the seed maturation stage in PVC tubes under normal (C) and drought stress (D) conditions.
(TIF)

S11 Fig Spatio-temporal expression patterns of *OsJAZ1* in various tissues. Data from the RiceXPro database. The expression level of *OsJAZ1* in root is indicated by a dashed box.
(TIF)

S12 Fig Root phenotypes of *OsJAZ1*-OE plants at the seedling stage. (A) Visual phenotype of *OsJAZ1-*OE plants and wild-type ZH11. (B) Seedling length, root length, and crown root number of the *OsJAZ1-*OE plants and wild-type ZH11. The data represent the mean ± SE (n = 15). **P < 0.01, Student’s *t*-test.
(TIF)

S13 Fig Overview of the PVC-tubes root phenotyping procedure. (A) Planting arrangement in the PVC tubes. (B) Rice plants in the PVC tubes. (C) Photo of the roots washed from PVC tubes. (D) Flow chart of the root trait investigation.
(TIF)

S1 Table Information about 529 accessions utilized in our study. (XLS)

S2 Table List of 413 suggestive associations in our study. (XLS)

S3 Table List of 264 suggestive loci in our study. (XLS)

S4 Table List of 73 co-localization loci by multiple traits. (XLS)

S5 Table List of 305 reported root related QTLs in our study. (XLS)

S6 Table List of 11 reported root related genes in regions of association loci. (XLS)

S7 Table Association and haplotype analysis of *Nal1*. (XLS)

S8 Table Association and haplotype analysis of *OsJAZ1*. (XLS)

S9 Table Genotype of IR64 type 1-bp deletion in *DRO1* in 529 accessions. (XLSX)

S10 Table List of 28 candidate genes in the association loci selected by bioinformatic analysis. The expression in root was regarded as "high" when its Cy3 signal intensity in root tissue in the RiceXPro database was higher than 10,000, otherwise it was regarded as "normal".
(XLS)

S11 Table Primers utilized in this study. (XLS)
