# Supplementary figures and images for: Genetic control of the root system in rice under normal and drought stress conditions by genome-wide association study
Source: PLoS Genet. 2017 Jul 7;13(7):e1006889. doi: 10.1371/journal.pgen.1006889 (PMC5521850; doi:10.1371/journal.pgen.1006889)

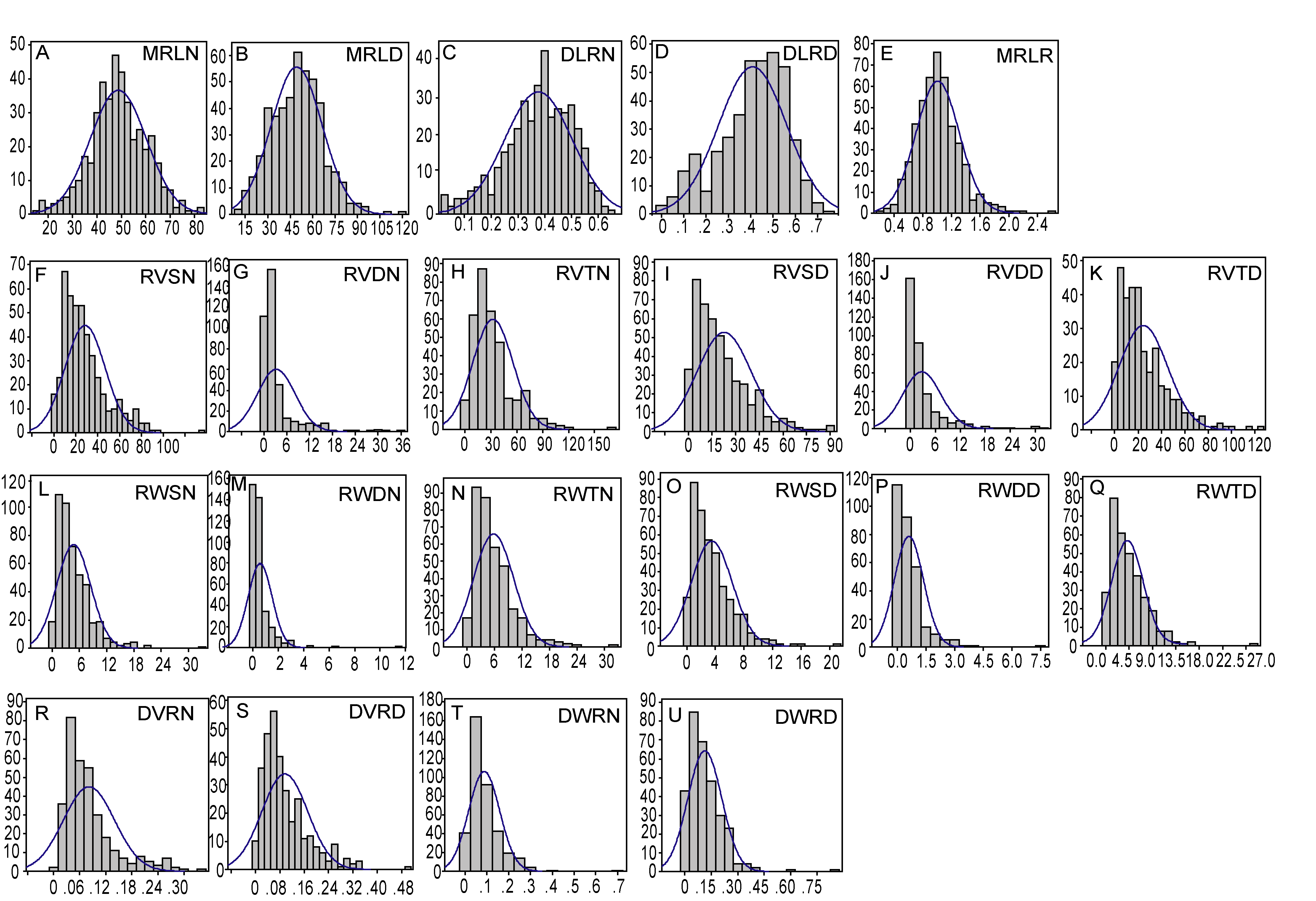

Supplement: S1 Fig — (TIF) [file pgen.1006889.s001.tif]

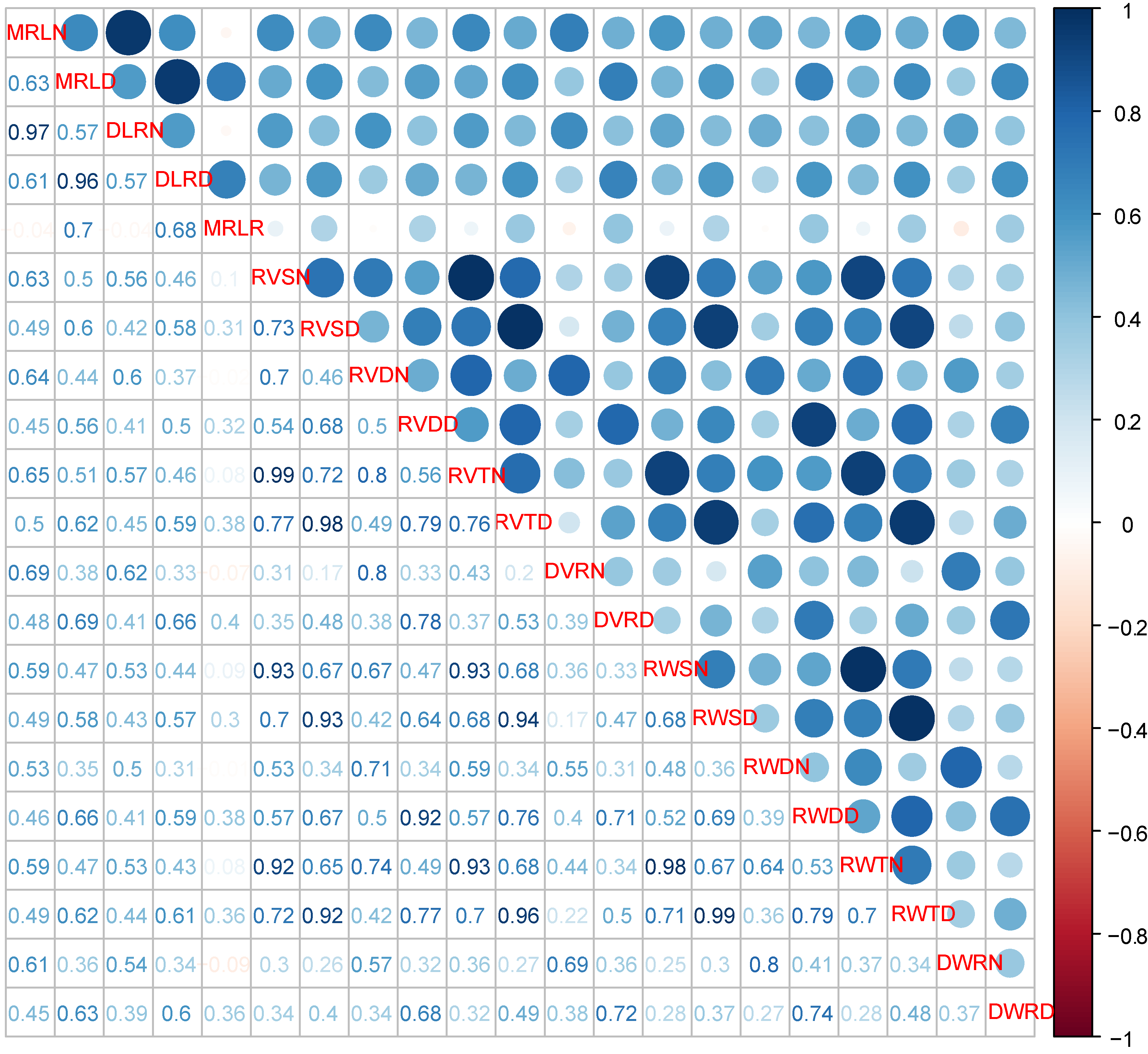

Supplement: S2 Fig — The correlation coefficients are shown in the lower left part of the figure. The correlation coefficients are indicated by the color (referring to the scale on the right) and size of the circles in the top right part of the figure. (TIF) [file pgen.1006889.s002.tif]

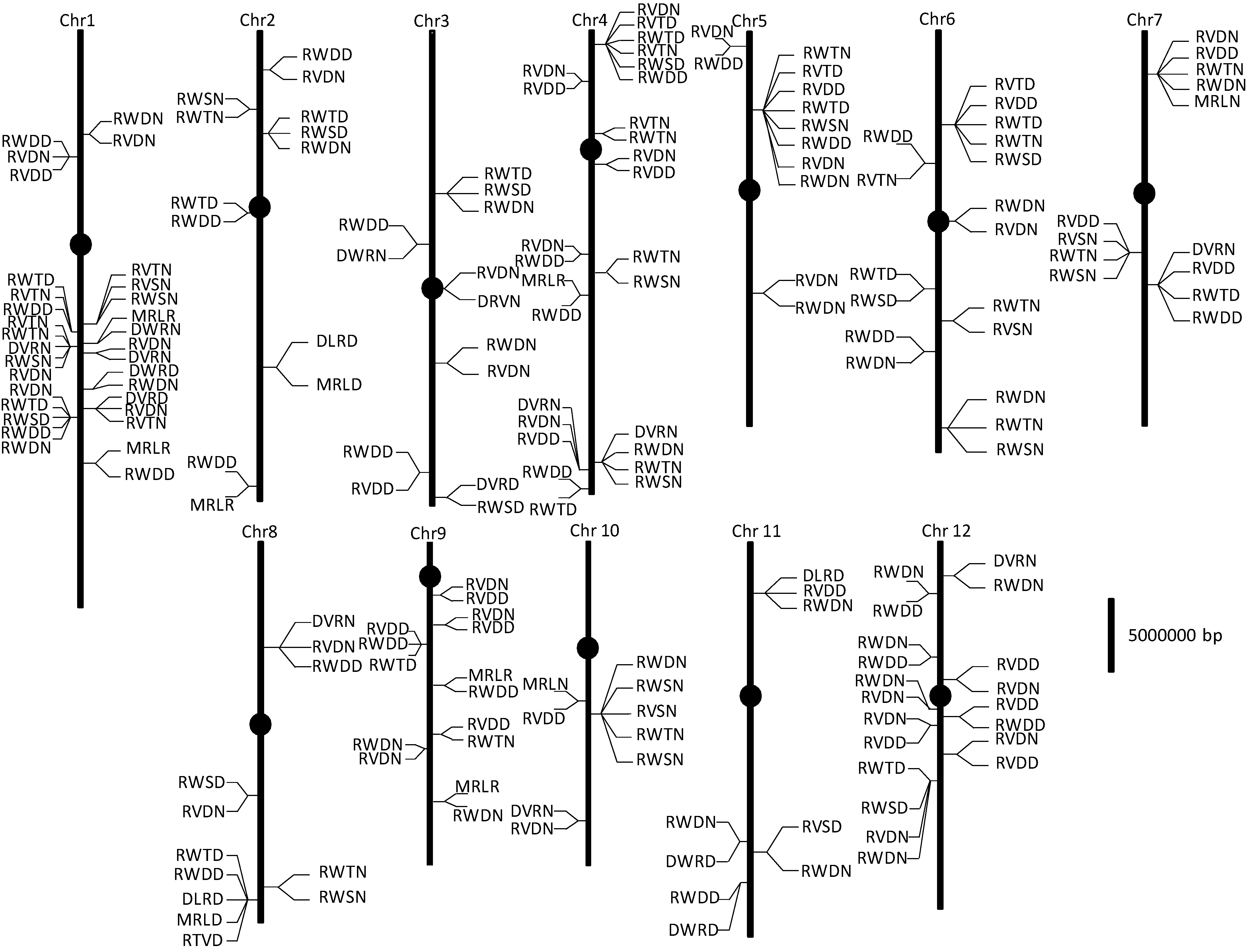

Supplement: S3 Fig — The vertical long bars represent chromosomes and black dots represent centromeres. Scale bar, 5 million base pair. (TIF) [file pgen.1006889.s003.tif]

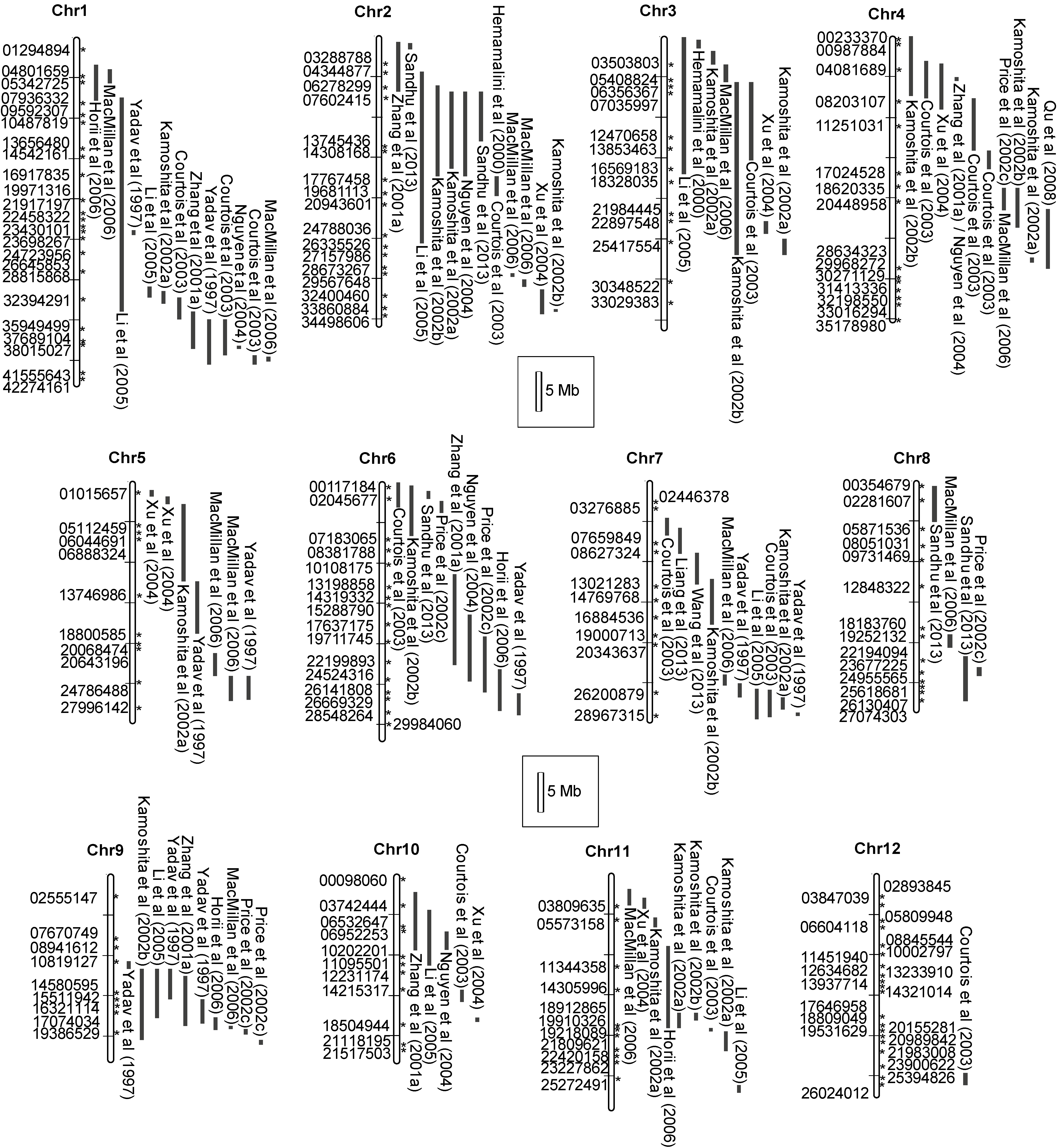

Supplement: S4 Fig — The vertical long bars represent chromosomes, the short black bars represent the QTLs for root weight, and the asterisks represent the association loci for root weight. The numbers corresponding to asterisks represent the lead SNPs for the association loci. Scale bar, 5 million base pair. (TIF) [file pgen.1006889.s004.tif]

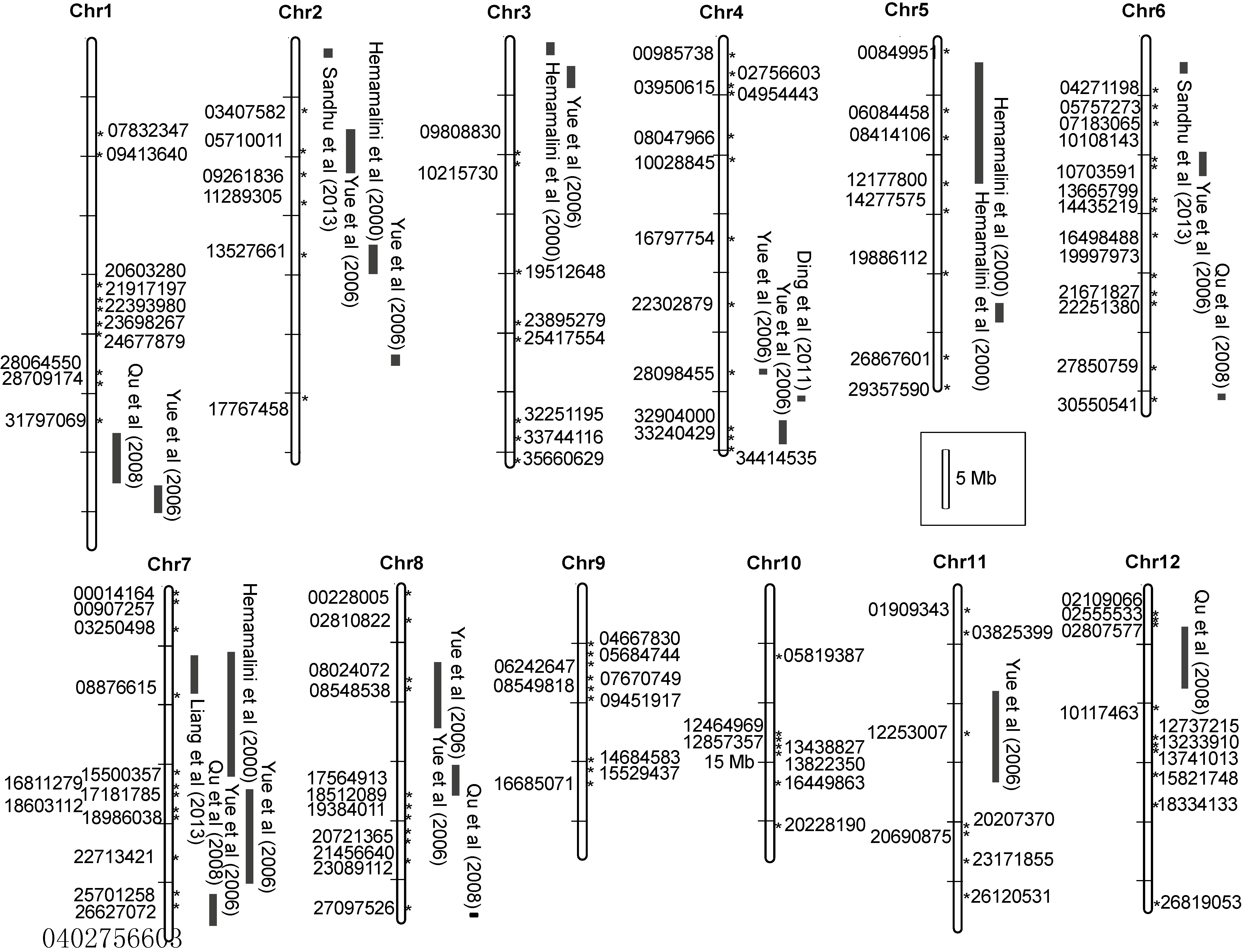

Supplement: S5 Fig — The vertical long bars represent chromosomes, the short black bars represent the QTLs for root volume, and the asterisks represent the association loci for root volume. The numbers corresponding to the asterisks represent the lead SNPs for the association loci. Scale bar, 5 million base pair. (TIF) [file pgen.1006889.s005.tif]

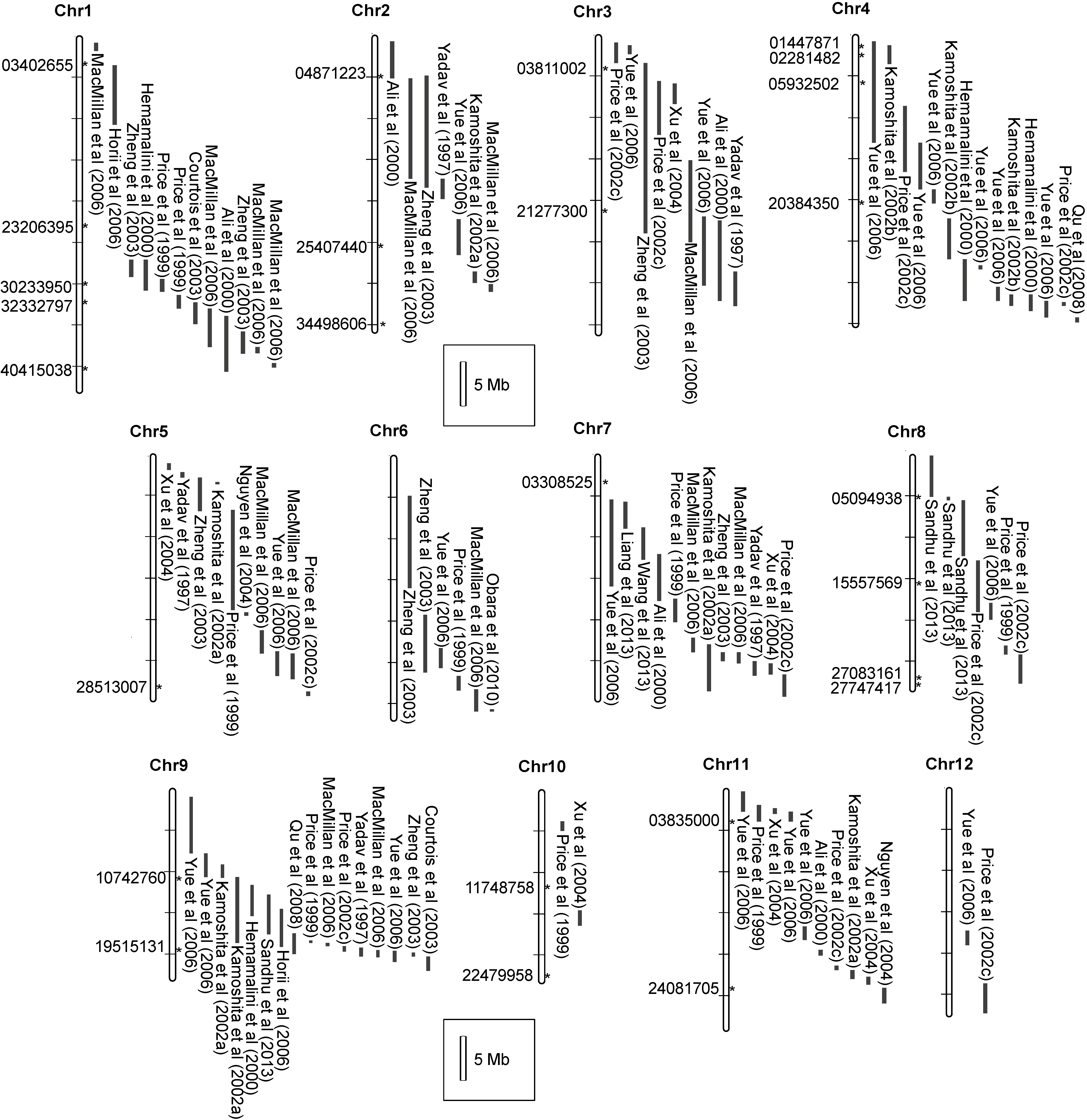

Supplement: S6 Fig — The vertical long bars represent chromosomes, the short black bars represent the QTLs for root length, and the asterisks represent the association loci for root length. The numbers corresponding to the asterisks represent the lead SNPs for the association loci. Scale bar, 5 million base pair. (TIF) [file pgen.1006889.s006.tif]

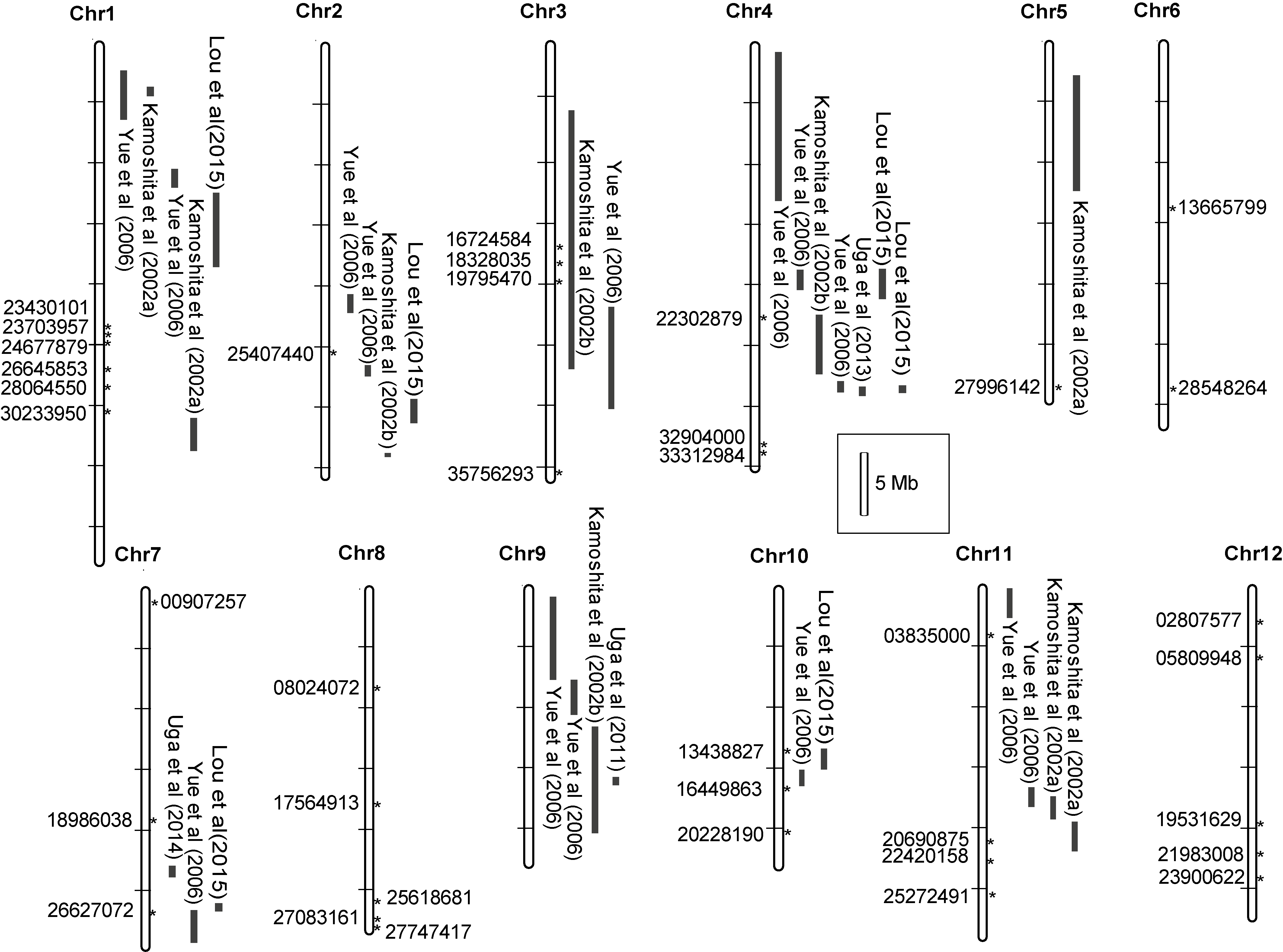

Supplement: S7 Fig — The vertical long bars represent chromosomes, the short black bars represent the QTLs for deep root rate, and the asterisks represent the association loci for deep root rate. The numbers corresponding to the asterisks represent the lead SNPs for the association loci. Scale bar, 5 million base pair. (TIF) [file pgen.1006889.s007.tif]

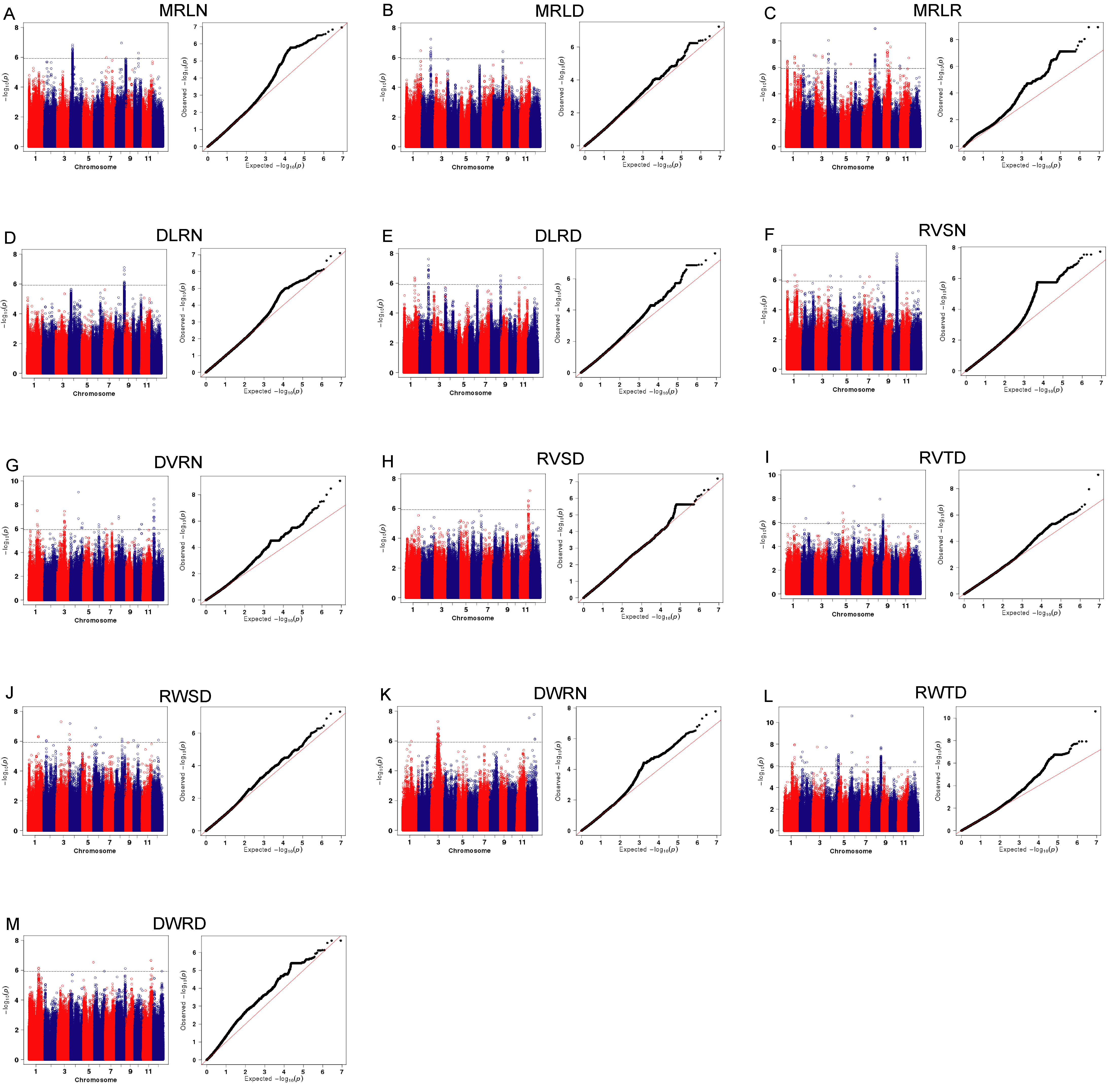

Supplement: S8 Fig — Manhattan plots (left) and quantile-quantile plots (right) are presented for (A) MRLN, (B) MRLD, (C) MRLR, (D) DRLN, (E) DRLD, (F) RVSN, (G) DVRN, (H) RVSD, (I) RVTD, (J) RWSD, (K) DWRN, (L) RWTD, and (M) DWRD. For the Manhattan plots, -log10 P-values from a genome-wide scan were plotted against the position of the SNPs on each of 12 chromosomes, and the horizontal grey dashed line indicates the suggestive threshold (P = 1.21×10^−6^). For the quantile-quantile plots, the horizontal axis indicates the -log10-transformed expected P-values, and the vertical axis indicates the -log10-transformed observed P-values. (TIF) [file pgen.1006889.s008.tif]

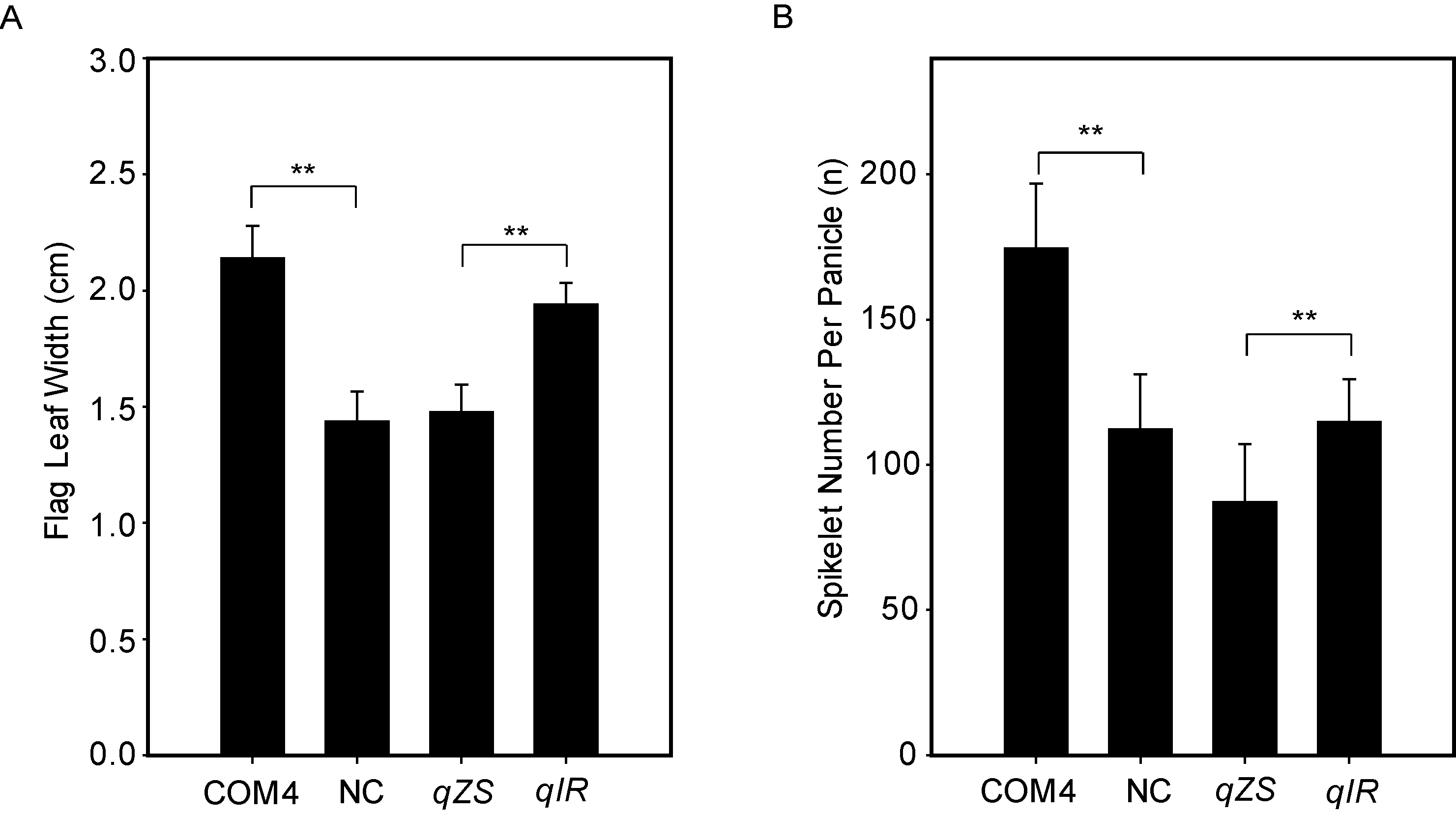

Supplement: S9 Fig — Flag leaf width (A) and spikelet number per panicle (B) of the complementary lines and NILs. The data represent the mean ± SE (n = 10). **P < 0.01, Student’s t-test. (TIF) [file pgen.1006889.s009.tif]

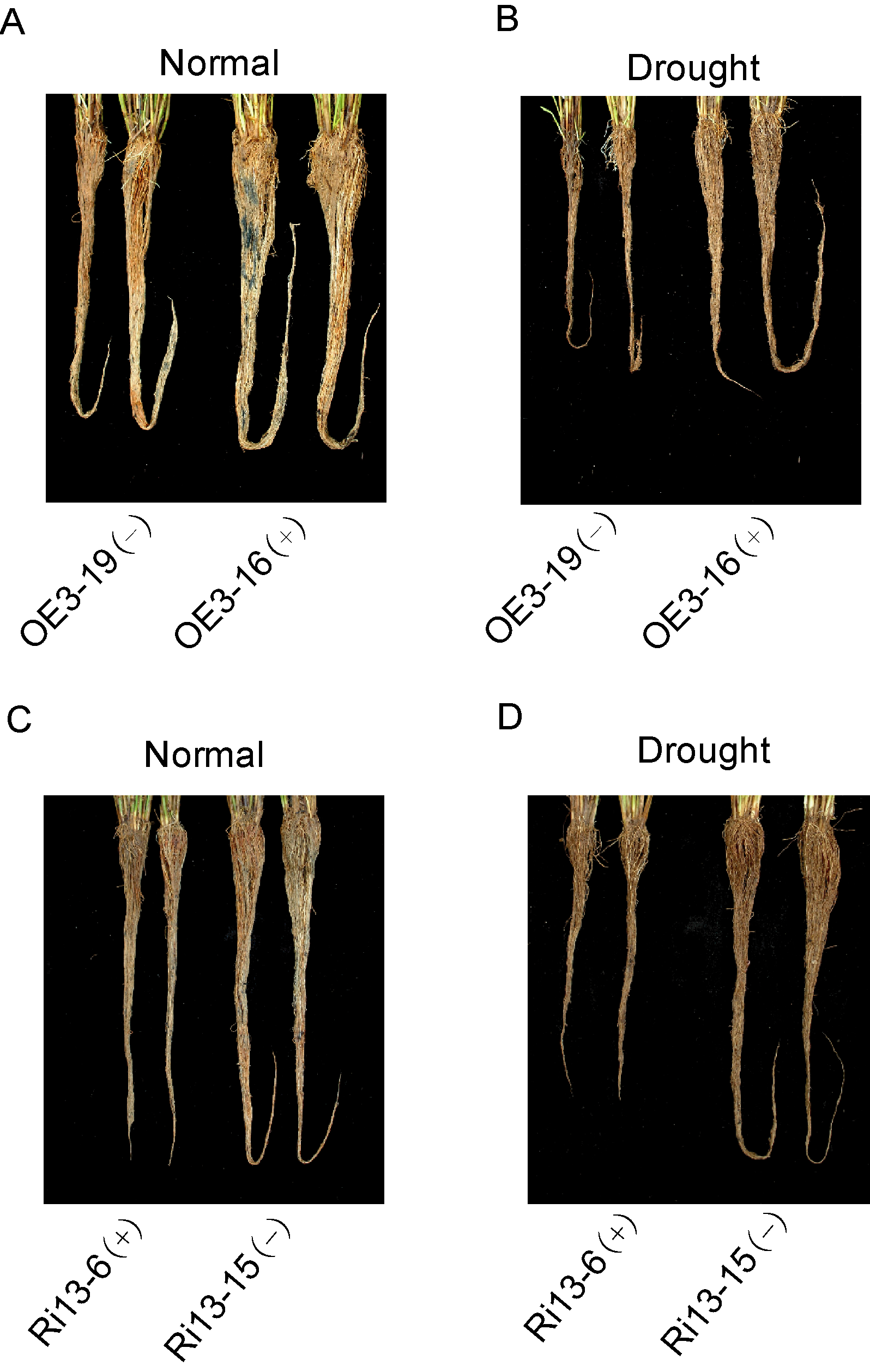

Supplement: S10 Fig — (A, B) Visual root phenotypes of the Nal1-overexpression (OE3-16(+)) plants and the segregated negative-transgenic control (OE3-19(-)) at the seed maturation stage in PVC tubes under normal (A) and drought stress (B) conditions. (C, D) Visual root phenotypes of the Nal1-RNAi (Ri13-6(+)) plants and the segregated negative-transgenic control (Ri13-15(-)) at the seed maturation stage in PVC tubes under normal (C) and drought stress (D) conditions. (TIF) [file pgen.1006889.s010.tif]

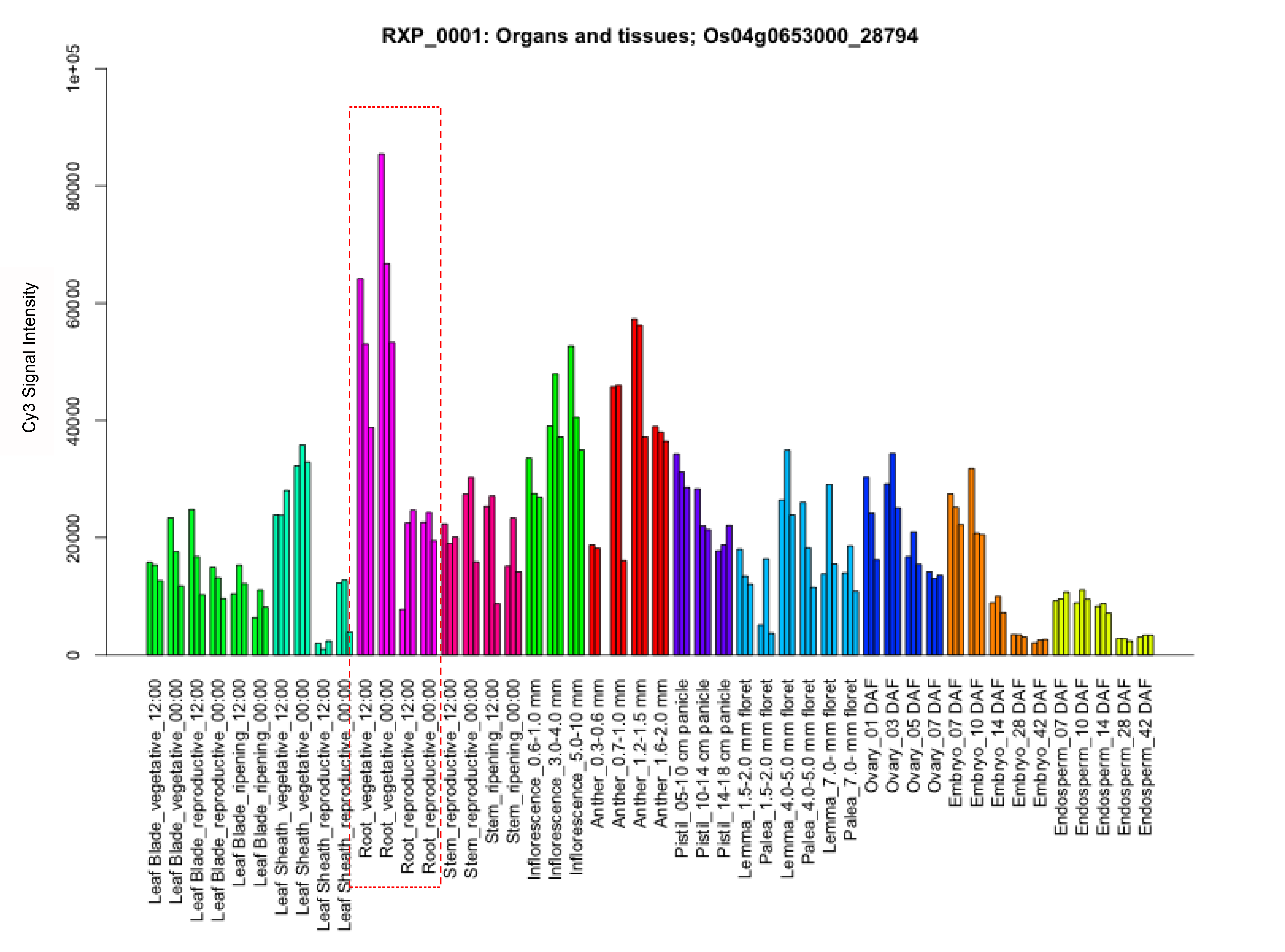

Supplement: S11 Fig — Data from the RiceXPro database. The expression level of OsJAZ1 in root is indicated by a dashed box. (TIF) [file pgen.1006889.s011.tif]

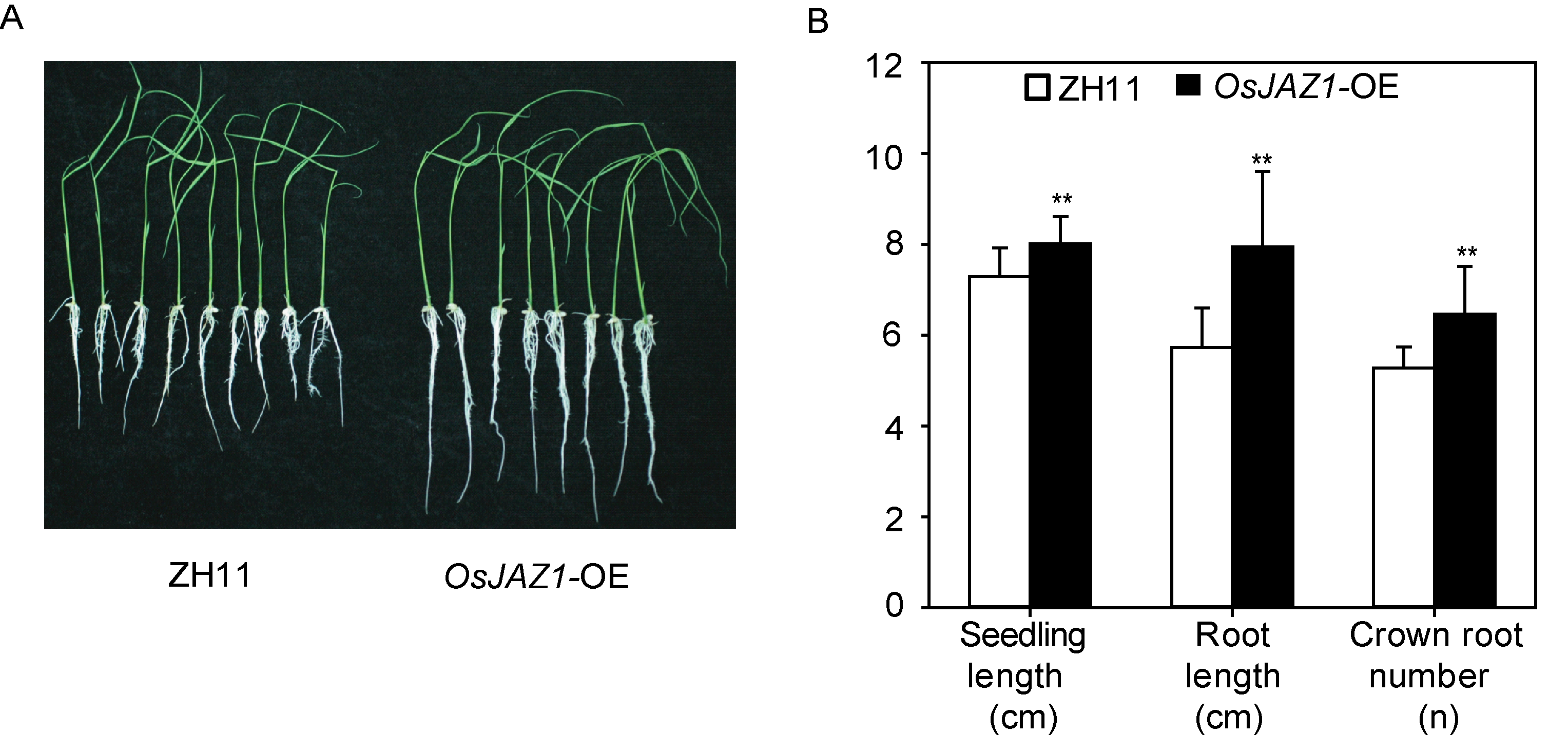

Supplement: S12 Fig — (A) Visual phenotype of OsJAZ1-OE plants and wild-type ZH11. (B) Seedling length, root length, and crown root number of the OsJAZ1-OE plants and wild-type ZH11. The data represent the mean ± SE (n = 15). **P < 0.01, Student’s t-test. (TIF) [file pgen.1006889.s012.tif]

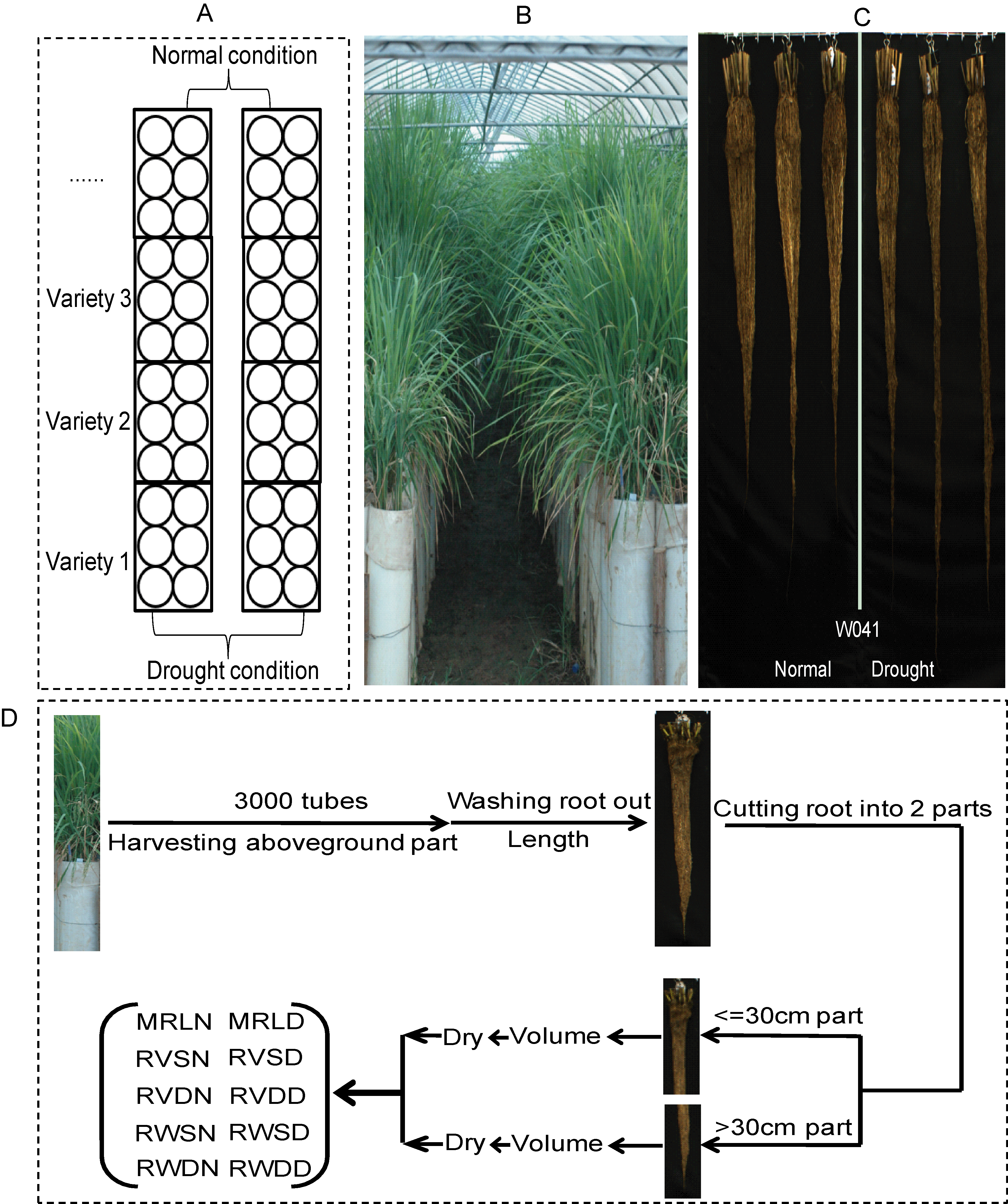

Supplement: S13 Fig — (A) Planting arrangement in the PVC tubes. (B) Rice plants in the PVC tubes. (C) Photo of the roots washed from PVC tubes. (D) Flow chart of the root trait investigation. (TIF) [file pgen.1006889.s013.tif]
